# Recent Advances in Bio-Based Flame Retardant Additives for Synthetic Polymeric Materials

**DOI:** 10.3390/polym11020224

**Published:** 2019-01-31

**Authors:** Christopher E. Hobbs

**Affiliations:** Department of Chemistry, Sam Houston State University, Huntsville, TX 77340, USA; chobbs@shsu.edu; Tel.: +1-936-294-3750

**Keywords:** flame retardant, bio-based, green chemistry

## Abstract

It would be difficult to imagine how modern life across the globe would operate in the absence of synthetic polymers. Although these materials (mostly in the form of plastics) have revolutionized our daily lives, there are consequences to their use, one of these being their high levels of flammability. For this reason, research into the development of flame retardant (FR) additives for these materials is of tremendous importance. However, many of the FRs prepared are problematic due to their negative impacts on human health and the environment. Furthermore, their preparations are neither green nor sustainable since they require typical organic synthetic processes that rely on fossil fuels. Because of this, the need to develop more sustainable and non-toxic options is vital. Many research groups have turned their attention to preparing new bio-based FR additives for synthetic polymers. This review explores some of the recent examples made in this field.

## 1. Introduction

Although instilling flame resistance has been a subject of interest since the ancient Egyptians [1], “modern” chemists have only been on the case since the 18th and 19th centuries, as evidenced by the words of American poet Emily Dickinson [2]: “Ashes denote that fire was—revere the grayest pile, for the departed creature’s sake, that hovered there awhile—fire exists the first in light, and then consolidates, only the chemist can disclose, into what carbonates.” The same era saw the publication of landmark work by French chemist Joseph Louis Gay-Lussac (who studied flame resistance with the intent of protecting theatres) and Scottish chemist M. M. Pattison Muir, who published The Chemistry of Fire in 1893, only a few years after Emily Dickinson’s death [3,4,5]. In the century-and-a-half since, our understanding of the molecular world, as it pertains to fire, has progressed significantly and the scientific community has made tremendous advances so that chemists can fully *disclose into what carbonates*.

**Figure 1:**
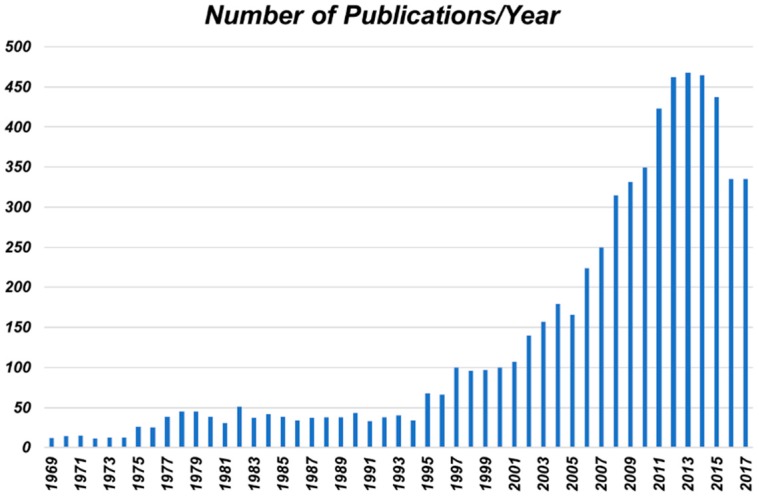
Graph representing the number of peer-reviewed journal articles (found in SciFinder) describing flame resistant (FR) polymers per year between 1969 and 2017.

The 20th century saw enormous advances in the chemical sciences. In particular, the rise of polymer chemistry led to a revolution in daily life across the industrialized world. Because of this, these synthetic materials (e.g., plastics and fibers) have become ubiquitous. However, the extremely high levels of flammability often exhibited by these materials is a consequence of their daily use. But, the advances made in polymer chemistry led to an increase in our understanding of fire resistance and provided chemists the motivation to study and develop better flame resistant (FR) polymers. Early commercial examples include: Bakelite, Nomex, Kevlar, and others. [3] This is understandably an important topic of industrial and academic research considering that structure fires cause $6.7 billion worth of damage, 12,300 injuries, and >2500 deaths annually in the United States alone [6]. The massive California wild fires in the summer and autumn of 2018 were a stark reminder of the threat that fires pose. For these reasons, the number of reports detailing the preparation of new FR polymeric materials has exploded in recent years (Figure 1). 

This growth in the number of reports of new FR materials has been spurred by advances in science as well as closer scrutiny by governmental regulatory bodies, many of which have sought to decrease and outright ban the use of halogenated FR materials in the last few decades [7]. In light of this, it has been the responsibility of the scientific community to develop new, safer alternatives to these traditional FR systems, of which phosphorus-containing species are some of the most studied [8]. To do so, it is important to understand how polymeric materials burn: combustion of polymeric material involves evolution of combustible volatiles through decomposition in an oxygen-rich atmosphere. No matter the type of FR used, the mechanisms for flame retardancy occur by one of two routes (or a combination of the two): (i) condensed phase or (ii) vapor phase [9]. Flame resistance/retardancy in the condensed phase often involves the formation of char layer that can stop the spread of the flame and self-extinguish, protecting the material by limiting the transfer of heat and oxygen to the polymer. Vapor phase flame resistance/retardancy involves terminating the radical chemistry involved in fire, often the mechanism by which traditional halogenated FR materials operate (Table 1). 

The chemical community has undergone an immense shift in our approach toward synthesis in recent years. This has been catalyzed by our increased cognizance of the environmental impact that our reliance on petroleum has had and the role it has played in global climate change. In addition, our current hydrocarbon feedstocks are renewable only on a geological timescale. So, chemists recognize and understand the need for more renewable and sustainable sources. This “Green Chemistry” revolution has impacted every aspect of chemistry, from biochemistry to synthesis [10]. The development of FRs is no different. In fact, one of the biggest recent objectives has been the synthesis and use of “bio-based” FR materials, which are more renewable and sustainable since they are not generated from our finite petroleum reserves [11]. Although there are hazards in working with any chemical (especially FRs that can decompose into toxic species upon combustion), the use of more renewable (bio-based) resources serves as a step in the right direction toward our realization of rendering the science more sustainable. In general, flame resistance can be introduced into polymeric materials through the incorporation of low molecular weight additives, either through blending or covalent attachment. The past several years has witnessed great growth in this field and many different bio-based materials have been used. The purpose of this review is to summarize recent advances made in this field. This review is organized around the bio-based material used and will mainly be focused on recent reports detailing the incorporation of such material into synthetic polymeric materials.

## 2. Bio-Based FR Materials

### 2.1. Tannic Acid and Related Materials

**Figure 2:**
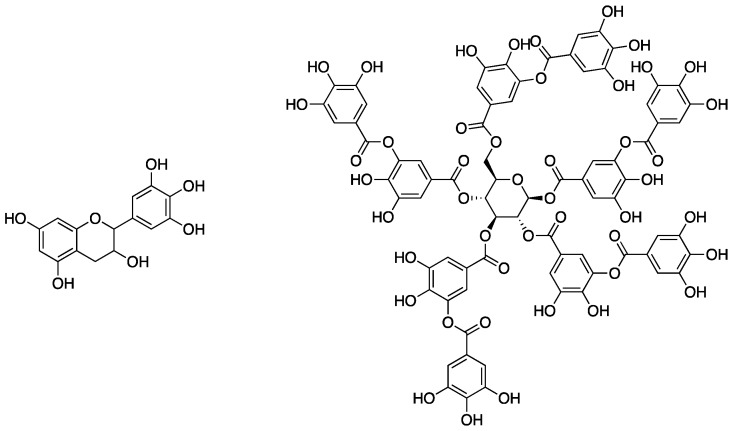
Molecular structures of a condensed tannin monomer (**left**) and a hydrolysable tannin (tannic acid, **right**).

Tannic acid is a member of the class of polyphenols, which are naturally occurring materials containing, as the name suggests, multiple phenolic moieties in their molecular structure (as can be seen by the structure of tannic acid in Figure 2). Some of the most abundant natural polyphenols are the tannins (both condensed and hydrolysable), which are found all throughout the plant kingdom. Tannins are the fourth most abundant material available from plants [12] and are utilized for antibacterial and insect repellent properties [13]. Despite their biological prevalence, the use of tannins as additives for synthetic polymers is relatively limited. This is possibly due to color or incompatibility with hydrophobic polymers [14]. However, tannins do exhibit excellent char-forming ability (due to the formation of cross-linked aromatic structures from the inner galloyl units during combustion) as well as antioxidant properties; these characteristics make tannins attractive targets for bio-based FR materials. In fact, these properties allow them to provide natural fire protection for trees [15].

Taking inspiration from Mother Nature, Tributsch and Fiechter [15] showed that naturally-occurring tannins could be used to instill synthetic polymers with higher levels of flame retardancy. To show this, the authors acquired tannins from the barks of quebracho, Canary Island pine (*Pinus canariensis*), and giant sequoia (*Sequoiadendron*) trees, by extraction into a water/acetone mixture using sonication. The obtained material was then mixed with a synthetic polymer (acrylonitrile-butadiene-styrene (ABS) resin), in which the thermal properties and combustion performance of bar specimens were tested and compared to various tree barks. Thermogravimetric analysis (TGA) under argon revealed that the tree barks displayed relatively high levels of thermostability up to 600 °C, leaving behind up to 60% carbonized solid. On the other hand, ABS resin underwent one step decomposition around 400 °C to leave behind ca. 2% charred residue. The thermal properties of the ABS could be affected by mixing with tannin, however. Mixing ABS with either 30 or 50 *w*/*w*% tannin resulted in materials that underwent slightly more complex decomposition (similar to tree barks) while leaving behind higher levels of charred remains—up to 27%. Similar results were noticed when ABS resin was mixed with various amounts of Canary Island pine bark instead of extracted tannin. The authors note that the inclusion of tannin increased the limiting oxygen index (LOI), but provided no values.

A few years later, Celzard and coworkers [13] showed that condensed tannin-formaldehyde polymeric foams could be used as superior flame retardants when compared to typical phenolic foams. Additionally, such materials are more attractive since tannins are relatively nontoxic and bio-based. They explained that the superior FR nature is due to the stable char formation. The materials described showed more difficult ignitions and lower heat release rates as well. The tannin-based foams tested showed heat release rates per unit area (HRRPUA) of 12 kW/m^2^, while pure phenolic and epoxy foams peaked at 106 and 314 kW/m^2^. Further, ignition times of tannin foams exceeded 100 s, while phenolic foams were 6 s. 

**Figure 3:**
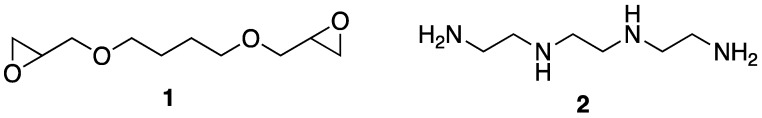
Molecular structures of 1,4-butanediol diglycidyl ether **1** and triethylenetetramine **2**.

In 2015, the laboratories of Wang and Schiraldi [16] reported on the incorporation of tannic acid into aerogels made of sodium montmorillonite clay and epoxy polymers. Advantageously, this preparation utilized relatively “green” procedures that involved mixing clay with 1,4-butanediol diglycidyl ether **1** and triethylenetetramine **2** (as a curing agent) and subjecting the mixture to freeze-drying in water (Figure 3). Incorporation of tannic acid could also be carried out by freeze-drying the same mixture with ca. 2% tannic acid. Further incorporation of tannic acid could be accomplished by coating the aerogels in water or ethanol solutions of either 2% tannic acid or 2% tannic acid-0.2% Ferric complexes. 

The FR properties of these materials were tested and described. The authors found that all aerogels exhibited some levels of self-extinguishing behavior when exposed to a propane torch for 30 s. Cone calorimetry provided peak heat release rate (PHRR), time to ignition (TTI), total heat release (THR), time to peak of heat release rate (TTPHRR), and fire growth rate (FIGRA). The results are summarized in Table 2 (in which the samples are named based on the percentage content of epoxy and clay, for example, a composite of 20% epoxy, 5% clay, and 2% tannic acid is E20C5T2). The authors note that typical PHRR of epoxy resins is ca. 1200 kW/m^2^ while the clay composite (even without the incorporation of tannic acid) exhibited values no higher than 400 kW/m^2^. Incorporation of tannic acid lowered this value by up to 20%. In addition to the flammability properties, the authors describe the differences in mechanical properties. This is an issue that may often be overlooked, but is important nonetheless; this is especially true if such materials have a future in commercialization. The authors noted that E20C5 aerogels were highly flexible and could recover ca. 95% of the original shape. The incorporation of only 2% tannic acid, however, resulted in a much more dense and stiff foam. Furthermore, even though flame retardancy could be enhanced by coating the samples in tannic acid/FeCl_3_ solutions, this resulted in a highly colored foam, which may not be aesthetically pleasing in a commercial sense.

Moustafa and coworkers [17] showed that the FR properties of tannic acid-ABS composites could be improved by the addition of CaCO_3_ (extracted from seashell wastes) as a bio-filler. Cone calorimetry showed that ABS containing 5% tannic acid displayed a TTI of 22 s and PHRR of 696 kW/m^2^, while the incorporation of 25% seashell waste into the composite exhibited a TTI value of 46 s and a 45% decrease in PHRR. Though the extent to which the amount of tannic acid effected the FR properties was not tested.

Inorganic hydroxides have been used as FR additives for a while now [18]. They are advantageous since they are relatively non-toxic and smokeless. However, polymer composites of metal hydroxides can exhibit poor adhesion to surfaces. Taking inspiration from mussels (in which catechol and amino groups are important for adhesion), it was recently shown that this problem could be addressed in a synergistic manner, by the incorporation of tannic acid-Fe(III) complexes and Mg(OH)_2_ into a polyamide-6 (PA 6, an industrially-important polymer) matrix [19]. Tannic acid-Fe complexes can be used to coat materials on the surface because of their strong chelating properties and were used in this study to stabilize polymer–hydroxide composites through non-covalent interactions. The composites were prepared by single-screw extrusion and studied using a variety of techniques. The authors showed that the incorporation of Mg(OH)_2_ into the PA 6 matrix could increase the LOI from 22.1% (0 wt % Mg(OH)_2_) to 29.1% (45 wt % Mg(OH)_2_). This could be improved with the addition of tannic acid–Fe complexes. For instance, the incorporation of 45 wt % Fe-complex and hydroxide led to an LOI of more than 31%. The authors note that in the absence of tannic acid–Fe complex, composites modified with 30 and 45 wt % hydroxide exhibited the same UL-94 classification. They concluded that there must exist a synergy between the metal hydroxide and tannic acid–Fe complex. Through TGA it was proposed that Mg(OH)_2_ and MgO (from decomposed magnesium hydroxide), along with Fe, facilitated the decomposition of tannic acid to form a protective char. Cone calorimetry was employed to further study this synergy. Unmodified PA 6 exhibited a PHRR of 512 kW/m^2^. This value could be reduced by 26% through the incorporation of Mg(OH)_2_ and tannic acid-Fe. Further, PA 6 left behind no residue while the Mg(OH)_2_/tannic acid-Fe composite left 32.4 wt % residue.

Considering that ca. 80% of deaths attributed to fires are actually caused by smoke inhalation [20], the reduction of smoke formation is an important aspect of FR. The authors studied the smoke production rate (SPR) and total smoke production (TSP) and found that the addition of 45 wt % Mg(OH)_2_ and tannic acid–Fe to the PA 6 matrix reduced the SPR from ca. 0.225 to 0.177 m^2^s^−1^ and reduced the TSP by 38% (1.307 m^2^). Notably, the inclusion of tannic acid–Fe(III) and Mg(OH)_2_ suppressed the formation of toxic gases as well.

**Figure 4:**
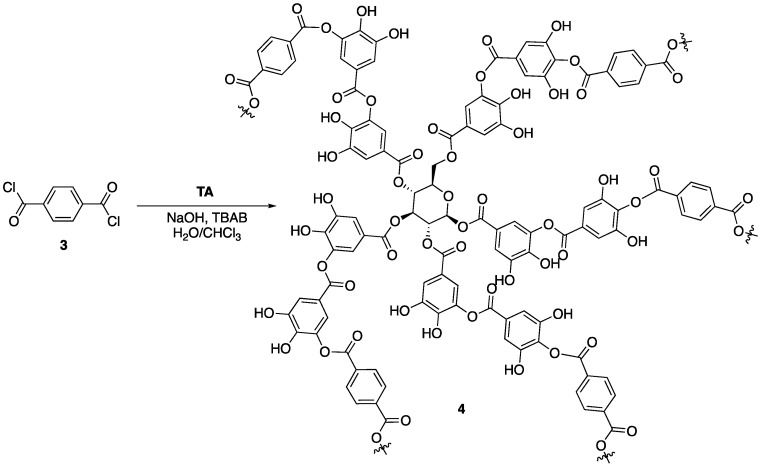
Preparation of tannic acid-terephthalate (TAT) **4**.

It has been shown that the thermal decomposition of tannic acid commences with the outer galloyl moieties to form pyrogallol and CO_2_ [21]. Mosurkal and Nagarajan [12] hypothesized that chemical modification and stabilization of the outer galloyl units would lead to enhanced FR properties of tannic acid. This was tested through reaction of tannic acid with terephthaloyl chloride **3** to form a complex network (tannic acid-terephthalate, TAT, **4**) that could be used as a FR coating for nylon fabric (Figure 4). First, the authors conducted a series of experiments aimed at deducing whether or not the modified tannic acid **4** exhibited enhanced thermal stability compared to tannic acid. The TGA analysis showed unmodified tannic acid to have a *T*_5%_ (temperature at which 5% mass loss occurred) to be 185 °C and a *T_max_* (temperature at which the maximum mass loss occurred) to be 333 °C, while the values for **4** were 299 and 531 °C, respectively. Additionally, the char yield of tannic acid was ca. 27% while **4** was slightly higher at ca. 37%. 

Upon completion of these studies, the authors prepared nylon samples treated with **4** by submerging nylon fabric in an aqueous suspension of **4** and acetic acid at 100 °C. The fabric was then washed, dried, and subjected to a modified ASTM D6413 vertical flame test. This test allows for the measurement of burning rate (determined by the height the flame reached within 10 s), afterflame time (i.e., amount of time recorded after ignition source is removed), and char length (distance between bottom of sample and furthest point the flame reached). The data are summarized in Table 3. As can be seen, nylon 66 coated with **4** showed enhanced FR properties when compared to nylon 66, both uncoated and coated with tannic acid. The two latter samples burned completely (127 mm was the length of the sample) and exhibited burning rates essentially double that of the **4**-treated samples. Furthermore, the **4**-treated nylon exhibited no melt dripping. Samples coated with tannic acid exhibited properties almost identical to that of the control. The authors note that this is because of the high-water solubility of tannic acid, which was washed out of the sample before the flame tests.

Polydopamine (PDA) is another attractive polyphenolic material and is environmentally friendly and non-toxic. Ellison et al. [22] reported on the use of mussel-inspired PDA-treated polyurethane (PU) foams recently. Furthermore, catechols have long been used as radical scavengers, an important mechanism of action for FR materials. The PU-dopamine composite foams were prepared by immersing PU foam in a buffered solution of dopamine hydrochloride. A series of thermal analyses and flame tests were then conducted. The TGA revealed that untreated PU (control) quickly reduced in weight and left no observable residue when heated to 600 °C, while pure polydopamine had almost 64 wt % residue at 600 °C. The PHRR and THR of control PU foam were found to be 498.3 W/g and 22.7 kJ/g, respectively. The PDA showed undetectable PHRR and a THR of 2.4 kJ/g. All PU foams were subjected to butane torch flame for 10 s to qualitatively study flammability. The control PU foam immediately ignited and formed a significant amount of PU melt pool; stopping this melt drip is important to reduce the spread of flame. The PDA-coated foams exhibited no melt dripping. The amount of PDA did improve the FR properties. The PDA1D (PDA coated for 1 day, 5.3 wt % PDA) underwent significant damage upon exposure to the flame while PDA3D (PDA coated for 3 days, 15.9 wt % PDA) greatly retained its shape and self-extinguished, protecting a sizable portion of the sample from the flame. Cone calorimetry was employed to further analyze the foams. Untreated PU foam showed a PHRR value of 734 kW/m^2^. Incorporation of 15.9 wt % PDA reduced this value to 239 kW/m^2^. Additionally, the authors showed that the generation of toxic gases (i.e., CO and CO_2_) were reduced from 0.053 kg/kg and 2.792 kg/kg, respectively, in the control foam to 0.013 and 0.028 kg/kg.

### 2.2. Phytic Acid

As mentioned earlier, one of the most vigorous areas of FR research has been the development of non-halogenated flame retardants. The reasoning behind this has been spurred by the environmental dangers that halogenated organics can pose to the environment. Furthermore, many government regulatory bodies have imposed strict regulations on their use. Some of the best performing replacements are phosphorus containing materials, either in polymeric form or as low molecular weight additives to synthetic or natural polymers [8]. One of the main mechanisms of action exhibited by phosphorus FR is their excellent ability to form protective char layers which affects self-extinguishing and protects the sample from the spread of the flame. There exists a plethora examples of phosphorus FR in the literature, many of which are outside the scope of this review and the author would encourage the reader to explore recent reviews [23,24,25,26,27,28].

**Figure 5:**
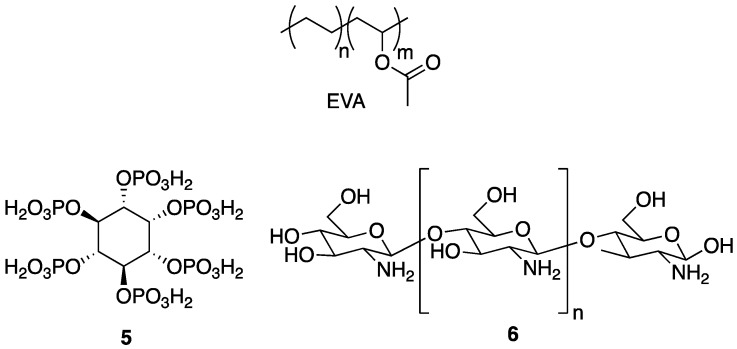
Molecular structures of ethylene-vinyl acetate (EVA), **5**, and **6**.

An issue at hand is the fact that many low molecular weight phosphorus species can be toxic. Although their toxicity could presumably be reduced through covalent attachment to a polymer, it may still be undesirable to work with such hazardous species. One safe, non-toxic alternative is phytic acid **5**, the structure of which is shown in Figure 5. Phytic acid **5** is a naturally occurring polyphosphate ester of inositol and is an important storage form of phosphorus in plant tissues [29]. Several research groups have taken advantage of this compound as a natural FR additive. In 2014, Zhang and coworkers [30] described the preparation of a polyelectrolyte complex (PEC) composed of a naturally occurring polymer, chitosan **6**, which is cationic under dilute acidic conditions (the structure of which is shown in Figure 5), and anionic **5** as a FR treatment for the synthetic copolymer of ethylene-vinyl acetate (EVA), an industrially-important material [30]. The PECs were prepared by the addition of aqueous solutions of anionic **5** into aqueous solutions of cationic **6**. The resulting material was incorporated into EVA composites (0, 5, 10, and 20 wt % PEC) by melt blending at 110 °C.

The authors first tested the thermal properties of **6** and the PEC by TGA. The *T*_5%_ and *T_max_* for **6** (control) were 271 and 298 °C, respectively. Chitosan **6** left behind char layer residue of 43 wt % at 600 °C. The PEC exhibited earlier decomposition, but the *T_max_* was 17 °C higher and the char residue at 600 °C was 56 wt %. This suggested enhancement of the thermal stability. The TGA analysis of the EVA–PEC composites revealed more complex degradation patterns for composites with higher content of PEC. Additionally, the higher the PEC content, the higher the residual mass (0 wt % for EVA and 12% for EVA containing 20 wt % PEC). Furthermore, PHRR decreased as the content of PEC increased (801 and 552 W/g for EVA and EVA containing 20 wt % PEC, respectively). In turn, the LOI values also increased: 20 and 22.9% for EVA and EVA containing 20 wt % PEC, respectively. During the UL-94 vertical burn test, only EVA composites of 20 wt % PEC reached V-2 rating.

**Figure 6:**
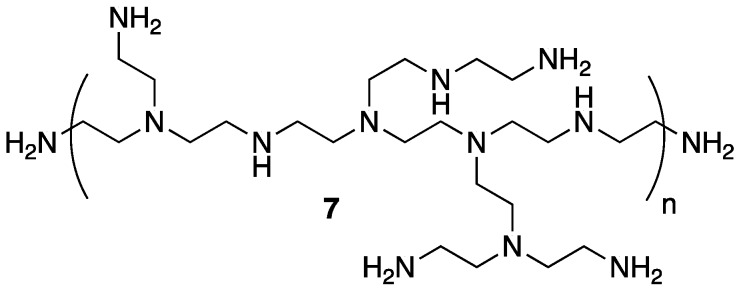
Molecular structure of **7**.

The same group applied a similar PEC system based on **5** and polyethylenimine **7** (Figure 6) as FR treatment for polypropylene (PP), one of the most widely used and important performance polymers [31]. The authors prepared PP composites of 5, 10, and 20 wt % PEC and compared their thermal stability to control PP, **5**, **7**, and composites of PP with the latter two. The TGA (summarized in Table 4) revealed a reduction in *T*_5%_ with the incorporation of PEC (due to the earlier degradation of PEC). The *T_max_* values increased as did char formation for PP–PEC composite. The authors note that this is due to the enhanced thermo-oxidative stability of PEC over PP alone.

The authors next tested the flammability properties of the samples using combustion calorimetry. It was revealed that the incorporation of PEC into the PP matrix could reduce the PHRR from 1129 to as low as 815 W/g (PP-20% PEC). This illustrated the synergistic relationship between **5** and **7**, as PP composites with **5** or **7** alone exhibited PHRR values of 938 and 965 W/g, respectively. Furthermore, LOI values were measured and a noticeable increase was observed between pristine PP, PP-5% PEC, PP-10% PEC, and PP-20% PEC (18.0%, 19.7%, 22.0%, and 25.1%, respectively). 

**Figure 7:**
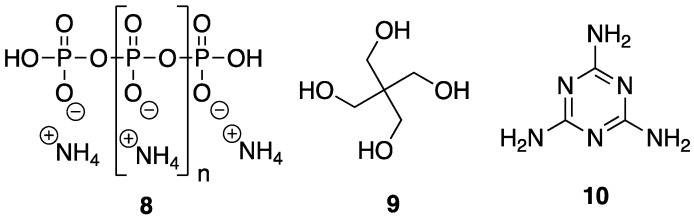
Chemical structures of **8**, **9**, and **10**.

**Figure 8:**
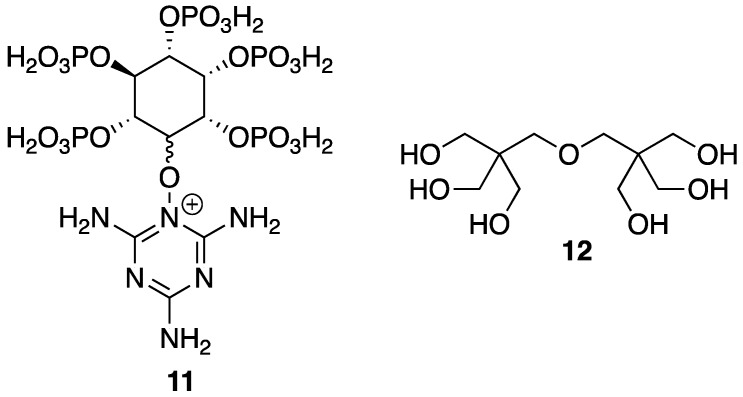
Proposed structure of **11** and structure of **12**.

In order to be considered an intumescent flame retardant (IFR), the material must contain an acid source, a blowing agent, and a charring agent. All of which are important for the formation of a dense layer of char foam upon combustion [32]. A common IFR used is based on ammonium polyphosphate **8**, pentaerythritol **9**, and melamine **10** (Figure 7). With the interest of green and sustainable chemistry in mind, Wang et al. [33] reported on the reaction between bio-based **5** and **10** to form a synergistic IFR additive for PP. The proposed structure (suggested from very limited spectroscopic techniques) of the resulting product **11** is shown in Figure 8. Product **11** was used as, both, an acid source and blowing agent and was used in conjunction with dipentaerythritol **12** as a charring agent. 

To gain insight into the thermal properties of these materials, the authors first employed TGA for the analysis of **11**, **12**, and a mixture of the two. Dipentaerythritol **12** underwent a one-step decomposition, beginning at 250 °C, to leave no residue. **11**, on the other hand, showed a more complex, three-step decomposition beginning with 15% weight loss between 250 and 350 °C, representative of release of ammonia and water. Dehydration followed between 350 and 600 °C to produce water and poly(phytic acid). Lastly, poly(phytic acid) decomposition occurs, generating phosphorus oxide (above 600 °C) to leave 25 wt % residue at 800 °C. Composites composed of PP were prepared by melt-mixing in a twin-screw extruder at 190 °C followed by hot-pressing under 10 MPa for 15 min. The LOI values of various formulations were measured and were found to increase upon increasing content of **11** and **12**. Control PP exhibited a value of 17% while the sample containing 7.5 and 22.5% **12** and **11**, respectively, exhibited an LOI of 28.5%. Furthermore, the latter obtained a V-0 rating in the UL-94 vertical burn test whereas the control PP exhibited extensive combustion and melt drip. The TGA experiments provided insight into the thermal properties of the composites. Unsurprisingly, control PP decomposed completely leaving behind no residue at 500, 600, and 700 °C. This could be increased, albeit slightly, to 6.16, 4.87, and 3.58%, respectively, upon the increase of **12** (7.5 wt %) and **11** (22.5 wt %) content.

**Figure 9:**
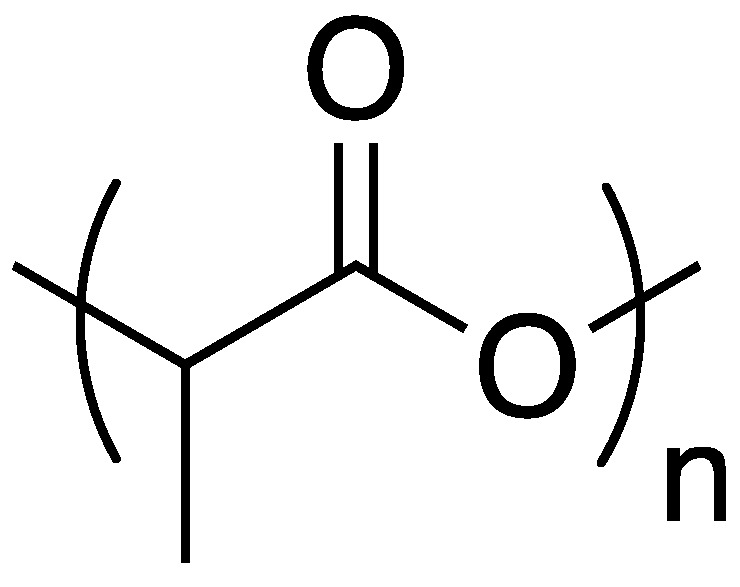
Structure of poly(lactic acid) (PLA).

Along with other sub-disciplines of chemistry, the ideas and principles underlying the philosophy of green chemistry have had a dramatic impact on the field of polymer chemistry. [34] In particular, the utilization of more sustainable resources for developing new materials or new methods to prepare existing materials is of tremendous importance. Considering the toll that society has taken on the environment, the preparation of biodegradable polymers is also important [35]. One of the most widely used biodegradable polymers is poly(lactic acid) (PLA, the structure of which is shown in Figure 9). Poly(lactic acid) can be prepared from vegetation (as opposed to petroleum) and breaks down into lactic acid, an environmentally benign product [35]. 

Although more sustainable, PLA suffers from the same detriments as many other plastics, it is highly flammable (much work has gone into the development of FR PLA [36], some of the studies utilizing bio-based FR for PLA will be described throughout this review). To solve this problem, Tang and coworkers [37] used **5** as a FR coating for PLA non-woven fabrics. The fabrics were prepared using melt-blown spinning technology and were treated with **5** simply by immersion into a PA solution (containing 50, 100, 150, 200 or 250 g of **5**) for ten minutes, followed by drying. The flammability of the dry, non-woven fabrics were then tested. A correlation of LOI with mass gain (essentially the amount of PLA absorbed) of each sample was observed. The LOI values of up to 36% were measured, for samples treated with 250 g of **5** (untreated PLA exhibited LOI of 26.3%). Vertical burn test revealed that none of the treated samples exhibited afterflame time and showed smaller burn area than the control. There was also a noticeable downward trend in PHRR, HRC, and THR between the control (465 W/g, 461 J/gK, and 19 kJ/g, respectively), PLA treated with 100 g of **5** (328 W/g, 327 J/gK, and 14 kJ/g, respectively), and PLA treated with 250 g of **5** (280 W/g, 278 J/gK, and 12 kJ/g, respectively). Temperature of maximum heat release (*T_max_*) also showed this trend for three samples, 375, 371, 365 °C, respectively. The TGA data was also provided and importantly showed an increase in the char residue of these three samples. While the control left behind no residue, PLA treated with 100 and 250 g of **5** left behind ca. 6 and 16% residue at 600 °C, respectively. Although an improvement over untreated PLA fabric, **5** on its own left 45% char residue at 600 °C. Further, the authors noted that the treated fabrics in this study were not durable as a few washes with water were all that were needed to remove most of the **5** from the samples.

A few years later Laoutid et al. [38] reported on the use of a combination of **5** and lignin as route for instilling FR properties to PLA. Lignin is a structurally-complex, highly crosslinked, naturally occurring polymer found in wood and bark. It has been used many times as a filler in polymer matrices to instill some FR properties, which it can do thanks to it aromatic structure that aids in char formation (FR polymeric materials using this bio-based material are outlined later in this review). The authors prepared polymer composites through melt processing PLA, **5**, and lignin in various proportions. The lignin samples used were prepared using either the kraft process or organosolv [39]. The authors first studied the thermal stability of the compositions as well as the additives alone. They noted that the two lignin samples exhibited different thermal behaviors, the details of which are outside the scope of this review. Suffice it to say, all additives exhibited char formation upon degradation. Combinations of lignin and **5** exhibited thermal decompositions completely different than the additives alone: a mixture composed of 50% lignin and 50% **5** exhibited a *T*_5%_ value much lower than any one of the components alone. This is possibly due to some dehydration processes that release low molecular weight components at lower temperatures. These mixtures also left char residues of ca. 50% at 800 °C. Next, the authors tested the thermal stabilities of PLA composites with lignin, PA, and both lignin and **5**. The PLA alone exhibited a one-step decomposition in TGA (*T_max_* = 371 °C) to leave no residue. The incorporation of lignin resulted in lower decomposition temperatures (20 wt % kaft and 20 wt % organosolv exhibited *T_max_* values of 332 and 271 °C, respectively) and higher char residue formation (5 and 11%, respectively). Phytic acid **5** exhibited similar results. Mixtures of lignin and **5**, however, resulted in TGA curves that closely resemble that of pristine PLA: PLA composite of 10% **5** and 10% organosolv lignin revealed *T_max_* of 381 °C, in which a synergistic effect of the PA and lignin are responsible.

Cone calorimetry was employed to study the FR properties with a heat flux of 35 kW/m^2^. The results are summarized in Table 5. As can be seen, pristine PLA readily ignites and burns completely to leave no residue. Incorporation of lignin seemed to increase the ignitability of PLA. Similar effects have been observed before and could be attributable to increase in heat absorption and earlier release of combustible products. Although the ignitability increased, the PHRR and THR both significantly decreased. The use of **5** as an additive alone had less of an effect on the TTI, but also led to decreases in PHRR and THR. Both lignin samples and PA led to higher char residue than the control PLA. Using PA and lignin in concert led to a TTI closer to that of PLA. Phytic acid **5** helps to reduce the thermo-degradation of PLA by lignin, as was suggested by TGA. Although the use of **5** alone did seem to improve the FR properties of PLA, the authors note that it is not ideal as it can leach from the polymer matrix within days. The use of PA in conjunction with lignin seemed to improve this. This synergistic effect manifested itself in scanning electron microscopy (SEM) as it seemed that the **5** helped in lignin particle dispersion within the PLA matrix. It was noted that this may ensure a more homogeneous formation of char upon combustion. Further analysis using UL-94 tests revealed that the PLA–PA composite as well as composite of PLA, **5**, and lignin samples gave V-2 ratings while the control (PLA) and PLA–lignin composites gave no classification. 

Lastly, although not directly related to the FR properties (but vastly important nonetheless), the authors stated that the incorporation of **5** and lignin had detrimental effects on the mechanical properties. Tensile strength for PLA was measured at 70.3 MPa. For example, incorporation of PA and Kraft lignin separately and together decreased this value to 27.7, 16.9, and 35.8 MPa, respectively. Elongation at break, as well as impact resistance, also decreased.

**Figure 10:**
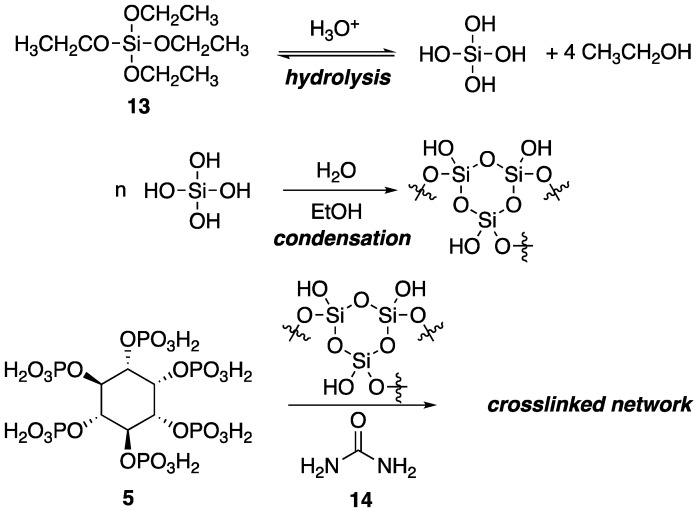
Sol-gel process to prepare **14**–**5** FR coating for polyacrylonitrile (PAN) fabrics.

Within the polymer industry, vinyl polymers make up a large bulk of the market, of which, polyacrylonitrile (PAN) is an important commodity in which acrylonitrile is often incorporated as a comonomer with monomers such as styrene [40]. Recently, Ren and coworkers [41] utilized a sol-gel process to prepare new FR organic–inorganic hybrid coatings that can be applied to PAN fabrics. Sol-gel processes usually involve the hydrolysis of reactive inorganic monomers in solution (“sol”) that can then condense to form crosslinked network (“gel”) that can be molded and shaped. The hydrolysis and subsequent reaction of tetraethoxysilane **13** is a classic example. In the current study, the authors applied this process to the preparation of a synergistic FR coating through the combination of **5**, urea **14** and **13** as a sol which was then used to coat PAN fabrics and allowed to gel (Figure 10).

The treated PAN fabrics were then analyzed to determine their thermal stability using TGA and were compared to untreated PAN fabric (control). The control sample exhibited a two-step decomposition: first, 32% weight loss between 276 and 366 °C resulting from cyclization-dehydrogenation reactions and a maximum weight loss of 24% between 366 and 550 °C. Carbonization occurred ca. 400 °C to leave behind a rather significant graphitic residue (ca. 44%). The PAN fabrics composed of Si, Si–**5**, and Si–**5**–**14** sol-gels were also tested. The PAN treated with the Si sol-gel exhibited thermal behavior similar to that of the control but showed a higher thermal stability (due to the presence of Si–O bonds) and enhanced char formation composed of SiO_2_. The PAN treated with Si–**5** sol-gel exhibited more complex behavior with 4 decomposition steps and exhibited early loss of water and lower onset temperature of cyclization (ca. 125 °C). This indicated that **5** promotes the cyclization of PAN. Maximum weight loss occurred at 324 °C. The lower decomposition temperature resulted in higher oxide char formations from the synergistic effect of the Si–O–Si and P–O–P bonds. This sample left a char residue of ca. 68% at 800 °C. The PAN fabric treated with Si–**5**–**14** sol-gel exhibited a four-step decomposition as well. Early weight loss (between 62 and 110 °C) of water and ethanol, followed by the weight loss up to 255 °C (and a maximum weight loss at 171 °C) is ascribed to the decomposition of **14**, which forms ammonia and can assist as a protective FR in the gas phase. Even though it left lower levels of char (ca. 56%), it does act as an effective FR. The LOI values for the control, Si, Si–PA, Si–**5**–**14** sol-gels are 18, 25.1, 30.3, 34.1%, respectively. Unfortunately, it was found that these values decreased significantly after each wash cycle (for example, LOI for PAN fabric coated with Si–**5**÷**14** sol-gel decreased to 23.4% after four washes).

Layer-by-layer (LbL) assembly has been shown to be a relatively rapid and efficient process to prepare multifunctional coatings on a variety of different substrates [42]. The coatings are formed through repeatedly depositing alternating layers of anionic and cationic materials. In 2018, Ji et al. [43] showed that LbL assembly could be combined with a sol-gel process to prepare flexible (many sol-gels become brittle) FR polyester composites. The sol-gel process was utilized to prepare a flexible polysiloxane (SSP) “sol” from methyltriethoxysilane (MTES). The LbL assembly was carried out by immersion of polyester fabric in the sol solution of SSP for 30 s, followed by washing with water. After drying, the sample was immersed in a PA solution (PAS) (for 0 to 20 min) and subsequently washed with water. Then it was immersed in SSP, washed, and dried. This process was carried out three times to yield a flexible polysiloxane coated polyester composite. The TGA showed that the amount of PA (controlled by varying the time the sample was immersed in PAS) could have a drastic effect on the thermal stability of the polymer samples. Pure polyester fabric showed a simple two-step decomposition to leave behind no residue. Soaking the sample in PAS for 5, 10, 15, and 20 min showed an increase in the residue after combustion to 17.3, 18.4, 18.5, and 21.5%, respectively. The *T_max_* was also affected and could be decreased to 439.8 °C (sample soaked for 20 min) from 441.3 °C (control). To gain more insight into the FR properties, LOI measurements and vertical flame tests were performed and the data is presented in Table 6. Upon ignition, the control sample burned completely and rapidly (burn rate of 1.19 mm/s) with an LOI of 21.6%. The authors noted that in all cases the spread of the flame was not stopped, but all samples burned to the top. However, the inclusion of more PA (as indicated by the soak time) did have some dramatic effects on the LOI values (up to 31.4%) and burning rates (down to 0.32 s). Further, the melt-dripping (a potentially devastating effect caused by house fires) could be reduced from 44% to 16.4%. Advantageously, the authors note that this LbL, sol-gel combination produced much more durable FR fabrics than treating a sample with PA alone. They showed that the amount of PA remained almost constant in most samples, even after 45 wash cycles. This was illustrated by LOI measurements after each wash cycle and it was shown that the worst-case scenario resulted in a decrease of only ca. 3%.

Another industrially-important vinyl polymer is poly(vinylchloride), or PVC. This material has become incredibly important and widely used for a variety of applications (for example, piping and flexible laboratory tubing) [44]. However, because of the presence of flammable plasticizers, flexible PVC can catch fire and burn rapidly [45]. 

Recently, Qu’s [46] lab showed that **5**-metal salts can be used as effective FR additives to PVC. The flexible PVC composites were prepared by mixing PVC with 40% dioctylphthalate (DOP, as plasticizer), 4% organic tin stabilizer, 0.5% calcium stearate, 0.5% stearic acid, and 15% PA–metal complex (either Zn, Cu, Al, or Sn). The melt was mixed, compressed into a sheet, and cooled. The FR properties of each sample were measured by cone calorimetry. The data is summarized in Table 7. The TTI showed little variation between PVC (control) and composites. On the other hand, LOI values could be increased from 24.9% (for the control) up to 30.3% for PVC-**5**-Sn composite. The inclusion of **5**-M complexes also had a significant effect on the protective char formation: control PVC burned to near completion while the **5**–Cu composite left ca. 19% residue. Furthermore, it was observed that PHRR could be decreased by almost 150 kW/m^2^ (from 329 to 181.77 kW/m^2^ for the control and **5**–Cu composite, respectively). Considering that most injuries and deaths attributable to fires is actually smoke inhalation, the total smoke production (TSP) of a FR material is very important and cannot be overlooked. As can be seen from Table 7, the TSP could be decreased from 42.20 to as low as 15.77 m^2^ for the control and Cu composite, respectively. 

### 2.3. Isosorbide

**Figure 11:**
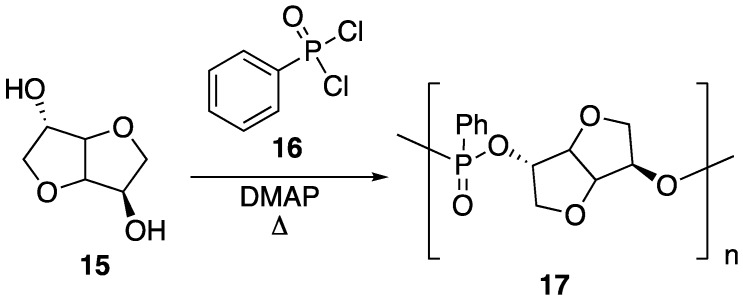
Synthesis of polyphosphonate **17**.

Isosorbide **15** is bicyclic diol that is can be obtained directly from D-sorbitol, which is ultimately derived from starch. This compound has, in recent years, attracted attention as a bio-based monomer for several polymeric materials [47]. Because of the presence of two hydroxyl groups, **15** can be instilled with various functionality. Recently, it has been shown that this can be taken advantage of for the preparation of new, bio-based phosphorus FR. Boday et al. [48] showed that a polyphosphonate **17** could be prepared through a stepwise, condensation polymerization reaction between **15** and phenylphosphonic dichloride **16** in the presence of *N*,*N*-dimethylaminopyridine (DMAP), according to Figure 11 [48]. Upon preparation, **17** (*M_w_* = 1020 g/mol) was blended with PLA (*M_w_* = 4032 g/mol) by solvent casting in chloroform, followed by removal of solvent under elevated temperature and reduced pressure.

Cone calorimetry was performed to obtain thermal stability information for blends of varying **17** content. Under a heat flux of 25 kW/m, little difference was observed between PHRR values of control PLA and PLA-**17** blends (PLA = 786, 5%, 10%, and 15% **17** = 796, 744, and 788 kW/m^2^). The THR was affected by the inclusion of **17**. Control PLA exhibited a value of 105 MJ/m^2^ while the 10% **17** blend showed a value of 82 MJ/m^2^. Further, the TTI decreased upon incorporation of **17**. To test the FR properties under more realistic conditions, the authors turned to UL-94 vertical flame tests. The PLA burned completely and could not be rated. PLA-**17** blends of 5, 10, and 15% **17** exhibited low extinguishing time (1.40, 0.80, and 0.10 s, respectively). The 15% **17** blend showed a V0 rating while the other blends were rated at V2. Unfortunately, the authors note that the increased content of **17** had deleterious effects on the mechanical properties. It was shown that the tensile strength and strain-at-break of the blends decreased 3-fold.

**Figure 12:**
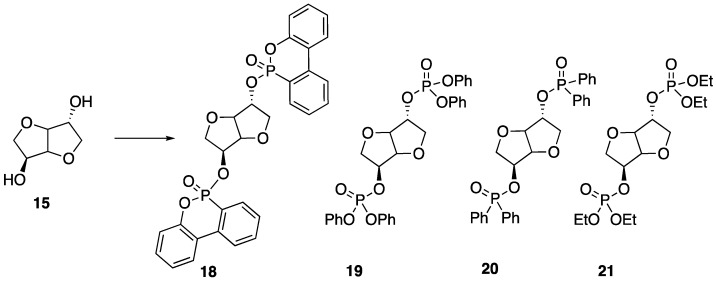
Isosorbide phosphate esters used as FR additives.

Recently, Howell and Daniel [49] were able to synthesize a variety of phosphate esters (**18**–**21**) from isosorbide that could be used as FR additives for epoxy resins (Figure 12). The phosphate esters could easily be prepared directly from isosorbide through reaction with the respective phosphoryl chloride. 

**Figure 13:**
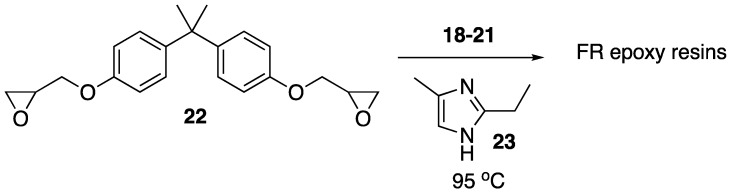
Preparation of FR epoxy resins from **22** and **23** containing **18**, **19**, **20**, or **21**.

Upon their synthesis, the authors studied the thermal stability of each derivative using TGA, which did not seem to reveal any clear trend between structure and thermal stability. However, the authors note that each compound revealed interesting isothermal degradation at 200 °C, in which phosphonate **18** seemed to be quite stable, losing less than 10% mass after 18 hours of heat exposure. Phosphonite **20** also exhibited some stability, decomposing by roughly 40% after 15 h of exposure. Phosphates **19** and **21** underwent relatively rapid decomposition to lose the corresponding phosphorus acid. In fact, all of the studied compounds lost phosphorus acid initially, just to different extents. Because of this, the authors hypothesized that such compounds may be in an effective condensed FR phase for epoxy resins. To test this, each compound was dissolved in bisphenol A diglycidyl ether **22** epoxy and then cured with 2-ethyl-4-methylimidazole **23** at 95 °C to provide resins with 1 or 2% phosphorus content (Figure 13). The thermal properties of each resin were then tested. The thermal stability of the resin was not dramatically affected by the presence of the additives with a few exceptions: the addition of **19** and **21** at loadings of 2 and 3.4% phosphorus provided onset temperatures of 329 and 319 °C in nitrogen, respectively (resin with no additive exhibited an onset temperature of 390 °C). The char formation could be increased from 8% (with no additive) to 26% (2% **20**). The LOI value for resin containing no additive was 18.8%. This could be increased to 31.6% upon the addition of 1% **20**. Fortunately, resins containing 1% **18** and **20** reached V1 rating while 2% **18** and **19** reached V0 in UL-94 vertical burn tests. 

### 2.4. Diphenolic Acid

**Figure 14:**
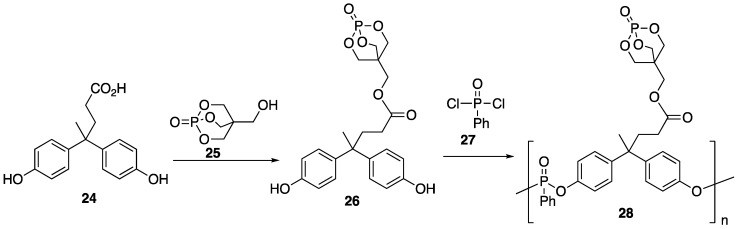
Preparation of **28**.

Diphenolic acid **24** is a bio-mass derived diol that can be prepared from reaction between phenol and levulinic acid, which can be produced from the degradation of cellulose [50]. Diphenolic acid **24** is an interesting structure because of the functionality that can potentially be incorporated into its framework through the hydroxy and carboxylic acids groups. Fang and coworkers [51] have taken advantage of this and prepared a novel bio-based polyphosphonante **27** that can be used as an effective FR additive for PLA. Polymer **28** could easily be prepared in two steps, beginning with reaction between **24** and 1-phosphabicyclo[2.2.2]octane-4-methanol **25** (to form **26**) followed by condensation polymerization with phenylphosphonic acid dichloride **27** (Figure 14). Blends of **28**/PLA were prepared in a melt mixer at 170 °C at 60 rpm for 8 min with 2, 4, and 6 wt % **28** to provide blends PLA 2, PLA 4, and PLA 6, respectively. 

The UL-94 vertical burn tests were performed and LOI values were measured. Pure PLA (control) exhibited an LOI of 20.0 while PLA 2, PLA 4, and PLA 6 were 28.8, 33.7, and 35.4, respectively. The UL-94 vertical burn tests of PLA (control) failed while PLA 2, PLA 4, and PLA 6 were classified as V2, V0, and V0, respectively. Cone calorimetry was used to study the combustion behavior of all samples. The data is summarized in Table 8. Pure PLA exhibited a PHRR of 418 kW/m^2^ and incorporation of up to 6% **28** only slightly decreased this value (388 kW/m^2^). Furthermore, TTI showed almost insignificant differences between the samples. The authors note this discrepancy between UL-94 and LOI tests and calorimetry and explained that these inconsistencies arise from the different fire scenarios recreated with each method.

Intrigued by the positive burn tests, the authors were prompted to test the FR mechanism at play. To do this, TGA was employed. Under N_2_ atmosphere, pure **28** showed a *T_onset_* of ca. 237 °C and left a char residue of 41.40 wt % at 600 °C. This sample exhibited two *T_max_* values (ca. 252 and 388 °C). Pure PLA exhibited a *T_onset_* of ca. 344 °C and a *T_max_* of ca. 379 °C with much lower residue (1.72 wt %) than **28**. The *T_onset_* of the PLA blends decreased with increasing **28**, as is common with phosphorus FR but showed no significant differences in *T_max_* values or residual char residue. The authors noted that these results, combined with the inconsistencies between experimental and theoretical residue, point toward a gas-phase FR mechanism. Additionally, TG-FTIR was utilized to study the evolved gases during thermal decomposition for **28**. At *T_onset_* (237 °C), a carbonyl stretch at 1775 cm^−1^ was observed, which indicates that the ester group was broken. At the first *T_max_* (252 °C), stretches associated with water (3660 cm^−1^), hydrocarbons (2970 cm^−1^), alkenes (1605 cm^−1^), ethers/alcohols (1101 cm^−1^), and P=O (1257 cm^−1^) were observed. Furthermore, at 388 °C signals associated with alkenyl C–H stretches were observed, indicating products evolved during char formation. The authors conclude that this serves as evidence that phosphorus-containing radical scavengers exist in the evolved gases. The PLA, on the other hand, showed water (3580 cm^−1^), hydrocarbons (3002–2850, 1400–1200 cm^−1^), CO_2_ (2341 cm^−1^), CO (2182 cm^−1^), and carbonyls (1761 cm^−1^). The PLA blends exhibited TG-FTIR spectra that were very similar to that of PLA, with the exception of a P=O stretch (1258 cm^−1^) for PLA 6.

A couple of years later, this same group described the application of **28** as an FR additive to PLA along with modified graphene oxide (M-GO) [52]. They chose this material because it has been shown to improve mechanical properties as well as impart some FR behavior because of its layered and graphitized structure. Because synergistic intumescent materials can be made with P and N-containing FR, graphene oxide (GO) was modified with **7** in the presence of 1-ethyl-3-(3-dimethylaminopropyl)carbodiimide (EDC). Composites were prepared through solution mixing of 80:20 ratio of PLA and M-GO in chloroform. The samples were dried and subjected to melt mixing with **28** at 160 °C to obtain composites containing 3 wt % of **28**/M-GO total with varying ratios of **28**:M-GO (3.0:0.0, 2.7:0.3, 2.4:0.6, and 2.1:0.9). The authors first tested the flammability of each sample. The results are summarized in Table 9. The LOI values were measured and compared to the control PLA. These values could be improved upon the addition of FR **28** and M-GO up to 35.6%. However, maintaining the amount of **28**:M-G at 3%, the LOI decreased as the amount of **28** decreased. Compared to PLA, all samples reached V0 or V2 ratings in the UL-94 burn test. The authors note that both samples containing no M-GO exhibited heavy dripping during the test. The incorporation of M-GO inhibited this dripping. The authors note that this was due to the increase in viscosity within the polymer matrix. Further, M-GO may slow the mass transfer in the condensed phase, leading to less dripping. Considering that **28** serves, mainly, as a gas-phase FR, the presence of M-GO can decrease the amount and speed of volatilization of decomposed gas product evolution. Cone calorimetry showed that each composite exhibited improved FR properties over PLA alone. The PHRR values could be decreased from 393 to 373 kW/m2 for PLA and PLA-2.1:0.9, respectively.

### 2.5. Deoxyribonucleic Acid (DNA)

As mentioned earlier, to be considered an IFR, materials must contain an acid source, charring agent (carbon source), and a blowing agent. Some of the most widely used and successful IFR materials are based on phosphorus and nitrogen-containing compounds. Some examples of these have already been discussed and may rely on a synergistic relationship between a P-compound (acid source, such as **8**) with an *N*-compound (blowing agent, such as **10**). However, preparing an IFR additive or polymer that contains both may be attractive. Even better would be using a naturally-occurring, bio-based compound that contains the acid source and blowing agent. Considering that its structure is based on nitrogenous base pairs, sugars and phosphate groups, deoxyribonucleic acid (DNA) contains all the elements required to act as an IFR. In fact, DNA has been used to instill FR properties to polymeric materials. Malucelli et al. [53,54] reported the first examples describing the utilization of DNA (extracted from Herring) as an FR coating for cotton fibers. 

That same laboratory then reported the first examples of DNA used in conjunction with synthetic polymers. This group described the preparation of FR ethylene vinyl acetate (EVA) composites utilizing DNA as an IFR additive [55]. The blends (containing 10, 15, and 20 wt % DNA) were prepared by melt-blending in a mixer at 120 °C and 60 rpm for 3 min. Once prepared, the authors analyzed the thermal stability of each sample using TGA. Under nitrogen, control EVA exhibited two degradation steps of maximum weight loss at 350 and 472 °C. Inclusion of 15% DNA reduced the onset temperature and exhibited a new *T_max_* at 240 °C from the decomposition of DNA. Importantly, it was shown that residue char formation at 600 °C increased with increasing DNA content: 10, 15, and 20 wt % DNA showed 6.0, 9.0, and 11.0% residue, respectively. Control EVA left behind less than 1.0% residue under nitrogen. The authors were also interested in investigating any synergistic effects of DNA with other carbon sources. They chose to study two other bio-based sources, α-cellulose (CEL) and *β*-cyclodextrin (*β*CD). The TGA showed that sample containing 10 wt % DNA and 10 wt % CEL (as well as samples containing 10 wt % DNA and 10 wt % *β*CD) exhibited behavior very similar to that of the sample containing 20% DNA. 

The FR properties were studied using cone calorimetry. Control EVA combustion was rapid with PHRR of 1588 kW/m^2^. The incorporation of DNA had a significant impact on this combustion. Although the TTI values decreased (from 65 to 28 s for EVA and EVA-10% DNA, respectively), the blends with DNA burned much more slowly and promoted the formation of protective char. Furthermore, the PHRR could significantly be reduced to 973 and 963 kW/m^2^ for the blends containing 10 and 20 wt % DNA. Although, the THR and TSR were almost the same as EVA alone. Blends of EVA, DNA, and CEL exhibited similar characteristics. Even LOI values for all blends were similar (between 20–22%). However, the sample containing 10 wt % DNA and 10 wt % CEL did show reduced CO and CO_2_ formation.

To improve on these results, the same laboratory later studied the differences in thermal and FR properties of impregnating versus simply coating the surface of EVA copolymers with DNA [56]. To coat the surface of EVA with DNA, DNA was deposited on EVA square plates followed by compression using a hot molding press at 120 °C and 5 MPa. Cone calorimetry was used to assess the resistance to heat flux of 35 kW/m^2^. As described earlier, the incorporation of DNA through melt blending reduced the TTI of EVA (from 62 to 28 s) and also showed a decrease of ca. 28% in the PHRR. Alternatively, when DNA is simply placed on the EVA surface, it takes up to 5 min for the sample to ignite. This corresponds to a 380% increase in TTI. This sample burned slowly with a PHRR of 348 kW/m^2^. The THR also decreased from 106 to 67 MJ/m^2^ for EVA–DNA melt blend and EVA coated with DNA, respectively. Furthermore, TSR was also reduced (from 1637 to 1520 m^2^/m^2^). These findings suggest that the protective char layer on the surface was able to protect the copolymer from combustion more efficiently than that of the sample prepared from melt-blending. The authors also studied each sample’s resistance to combustion using a burning-through test. Each sample was placed in a vertical position and flame from a butane lighter was applied three consecutive times. Each time the temperature of the sample was measured; it was found that EVA alone reached a maximum of 346 °C upon the third flame application while surface-modified EVA never surpassed 60 °C. Furthermore, the sample prepared using melt blending reached 188 °C.

Alongi [57] argued that this surface protection method could be a viable alternative to the bulk inclusion of FR additives to polymers and that any polymeric surface could, in theory, be coated without affecting the bulk polymer’s properties. To test this, Alongi’s laboratory applied DNA coatings to a variety of polymeric materials and measured their thermal stability as well as FR properties. Samples of polypropylene (PP), acrylonitrile-butadiene-stryene (ABS) resin, poly(ethyleneterephthalate) (PET), and polyamide 6 (PA6) were coated with DNA using hot compression molding press at 120 °C for 1.5 min. Each sample had a thickness of 3 mm and contained 10 wt % DNA. Cone calorimetry was employed to test the behavior of each material at heat flux of 35 and 50 kW/m^2^. In every case it was shown that PHRR could be significantly reduced while TTI values increased, similar to what was observed for EVA (Table 10). For example, PP shows a ca. 50% decrease in PHRR under either 35 or 50 kW/m^2^ heat flux.

**Figure 15:**
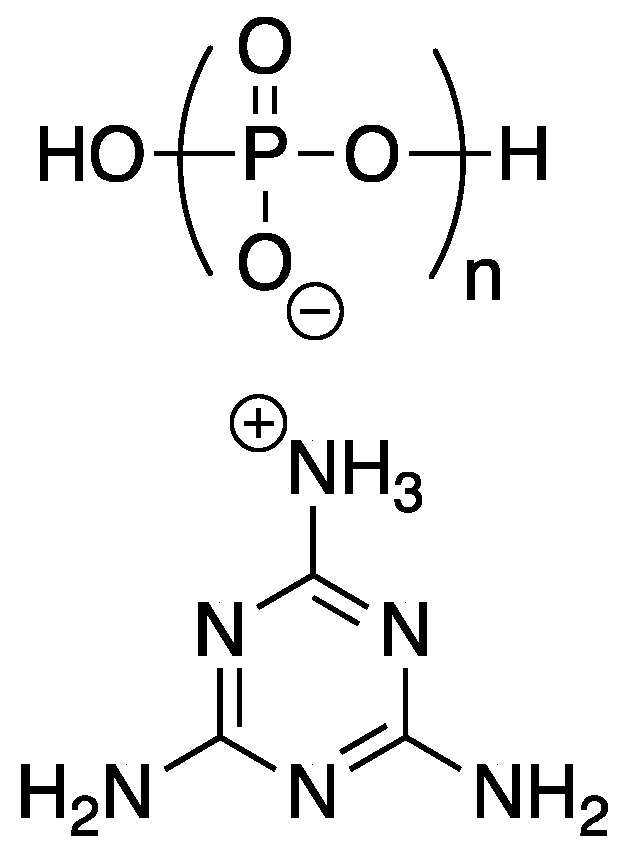
Chemical structure of **29**.

Pokorski and coworkers [58] were able to prepare FR low density polyethylene (LDPE, one of the most widely used polymers worldwide)–DNA composites through melt-blending. Additionally, the authors compared all thermal data to LDPE composites of melamine-polyphosphate **29**, a common industrial FR, the structure of which is shown in Figure 15. 

Thermal analysis was performed using TGA. Under nitrogen, LDPE undergoes complete decomposition at ca. 433 °C to leave no char residue. Samples of LDPE–DNA and LDPE–**29** blends (at loadings of 5, 10, 20, 30, and 40 wt % DNA or **29**) exhibited two major weight loss events: for DNA blends the first occurred around 200 °C and for **29** around 350 °C (due to decomposition of additive) while the second event occurred around 430 °C for both (due to LDPE decomposition). Char residue yields for pure DNA and **29** were 41.7 and 31%, respectively. The blends exhibited char yields from 1.3 and 2% (5% DNA or **29**) up to 11.4 and 12.1% (40% DNA or **29**). Interestingly, mico-combustion calorimetry (MCC) showed that neither DNA nor **29** caused any dramatic change in PHR, all values hovered around 500 °C. The PHHR (W/g) of LDPE was 1123.4 and could be decreased with increasing DNA or **29** content (695.5 and 701.6 W/g for 40 wt % DNA and **29**, respectively). The UL-94 vertical burn tests revealed FR properties of the polymer blends. It showed that the LDPE–DNA blends exhibited significantly reduced burn distance compared to **29** blends. This self-extinguishing is probably due to the early DNA decomposition. Additionally, it acts as a carbon source to facilitate char formation.

In 2017, Li and coworkers [59] described the preparation and use of a synergistic FR additive based on nitrogenous bases (the major components of DNA) and IFR system (composed of APP and pentaerythritol **9**). The PP composites were prepared by melt blending PP, nitrogenous base, and IFR. The samples were then analyzed by UL-94 vertical burn testing. Pure PP exhibited, unexpectedly, rapid ignition and heavy dripping (no classification) with an LOI value of 17.5%. The PP composite with IFR (18 wt %) increased the LOI to 24.5% but also could not be classified. At 25 wt % a V0 rating could be obtained with an LOI value of 28.3%. However, these exhibited some melt dripping. Incorporation of nitrogenous bases could, in some instances, improve the FR properties. For instance, a PP composite of 17 wt % IFR and 1 wt % cytosine (C) showed no improvement over the LOI value (27.9%) and had the same V0 rating, but melt dripping was not observable. Combustion behavior was further studied using cone calorimetry. Pure PP exhibited a PHRR value of 888 kW/m2 while the incorporation of 18 wt % IFR reduced this value greater than two-fold. This could further be improved to 324 and 293 kW/m^2^ through the inclusion of 1 wt % guanine (G) and uracil (U), respectively. 

### 2.6. Lignin

As noted earlier, lignin is a structurally-complex, highly crosslinked, naturally occurring phenolic polymer found in wood and bark. The exact chemical structure of lignin can vary dramatically based on the species (and part) of the plant from which it is extracted. Lignin is among the most abundant polymers in plant materials and is an abundant byproduct of paper production (approximately 50,000,000 tons of this material are produced each year). But, its variation in structure and amorphous composition has historically limited its use in valuable industrial processes (at the end of the 20th century, only ca. 1% of lignin produced was used in this manner) [60]. However, the highly aromatic nature of lignin structures allows for high levels of char formation upon combustion. For this reason, the use of lignin as a bio-based FR and antioxidative additive for synthetic polymers has been investigated since the early 2000s. In addition to its char formation, its antioxidative activity can be attributed to its high reactivity towards free radicals.

**Figure 16:**
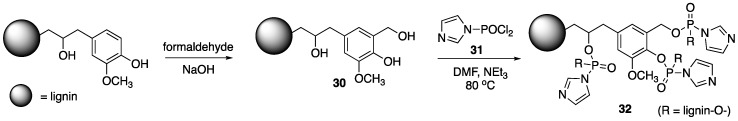
Preparation of **32**.

Song and coworkers [61] described the preparation of a chemically-modified lignin derivative. Commercially-available lignin (isolated from wheat straw) was grafted with nitrogen and phosphorus-containing compounds to improve the intumescent properties. This was achieved by first treating lignin with formaldehyde in the presence of NaOH (aq). This hydroxymethylated lignin **30** was then subjected to reaction with the **31** to form **32** (Figure 16). This material was characterized by IR spectroscopy and XPS. 

Before this material was used as an FR additive, its thermal stability was first tested. The TGA showed that unmodified lignin (control) started to decompose at ca. 250 °C. A maximum weight loss (*T_max_*) was exhibited at 405 °C and a char residue of ca. 41% was left behind at 600 °C. The TGA revealed **30** to have slightly higher stability with a char residue of ca. 51%. Modified lignin **32** exhibited an initial degradation (5% weight loss) at 240 °C, with a more complex degradation pathway, leaving behind almost 62% char residue. The ability of this material to act as an FR additive was then tested. Modified lignin **32** was incorporated into poly(propylene-*co*-ethylene) (PPE) samples using melt blending. The thermal stabilities of these composites were then analyzed using TGA. Virgin PPE (control) exhibited initial decomposition (*T_i_*) and *T_max_* values of 324 and 418 °C and left no char residue. Incorporation of 20 and 30 wt % of **32** to PPE lead to increases in *T_i_* (325 and 361 °C, respectively) and *T_max_* (479 and 483 °C, respectively). These higher values are related to the higher char formations. The sample containing 20 wt % **32** left behind ca. 12% char. This value was comparable to polymer blends with 30 wt % pure lignin, showing that the incorporation of P and N can increase the thermal stability and char formation. Cone calorimetry was used to measure the FR properties of these materials. It was shown that virgin PPE burns rapidly with a time to ignition (*t_ign_*) of 49 s, PHRR of 1350 kW/m^2^, and THR of 87.3 MJ. Incorporation of 20 wt % **32** exhibited *t_ign_* of 38 s, PHRR of 380 kW/m^2^, and THR of 74.2 MJ while 30 wt % PN-lignin incorporation values were 39, 360, and 69.7. Both cases revealed dramatic decreases in PHRR (ca. 70% decrease) and THR. 

**Figure 17:**
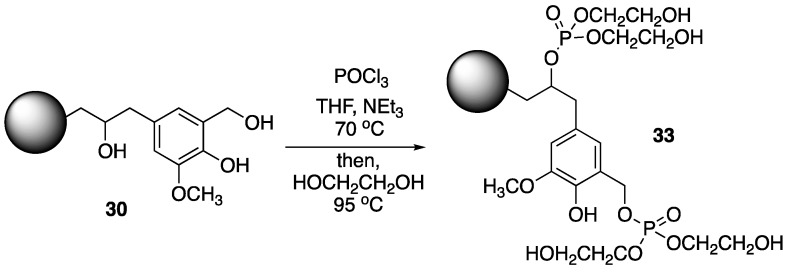
Preparation of lignin **33**.

Hu et al. [62] used a similar strategy for the preparation of synergistic FR systems based on chemically-modified lignin PU foams and ammonium polyphosphate **8**. Wheat straw lignin was first converted to **30**, which was then treated with phosphoryl chloride, followed by ethylene glycol to form modified lignin **33** (Figure 17). The PU foams were prepared by mixing **33** and PFAPP (microencapsulated **8**, prepared from reaction with phenol, formaldehyde and **8**) with methylene diphenyl diisocyante (MDI) and polyol in various amounts. The thermal behavior of the resulting materials was then investigated.

The TGA showed that pure PU exhibited first decomposition at 250 °C followed by second stage at 460–670 °C with no char residue. Analysis suggests that incorporation of PFAPP and **33** improved the PU foam stability: initial decomposition was increased to 278, 285, and 289 °C for composites containing 10, 20, and 30% modified lignin, respectively. Furthermore, these composites exhibited much higher levels of char residue upon combustion (28.1, 29.2, and 42.7%, respectively). Flammability properties were tested by LOI and cone calorimetry. Incorporation of 10, 20, and 30% **33** increased the LOI values to 23, 23.5, and 24.5% from 20% for pure PU. The PHRR values were dramatically reduced from 401 KW/m^2^ (pure PU) to 229, 193, and 165 KW/m^2^ for these samples. This noted decrease was caused from the protective char formation that occurs in the presence of modified lignin and **8**. 

The covalent modification of the PU foam is important since simply adding typical FR materials to PU foams can result in dramatic mechanical deterioration. This was further illustrated by Chen, Wan, and coworkers [63] in 2014 in which they described another method of PU foam preparation using a lignin-based phosphate–melamine compound (LPMC). This material was chosen because phosphate-melamines are traditional intumescent FR. Lignin (obtained from corn straw used in ethanol production) was first subjected to liquefaction with sodium lignosulfonate and H_2_SO_4_ in a mixed solvent system of glycerin and low-molecular weight (ca. 400 Da) polyethylene glycol (PEG) at 160 °C. AlCl_4_ was added, followed by polyphosphoric acid (PPA), to perform the esterification. This was followed by the addition of melamine. Upon isolation, PU foams of varying LPMC content (0, 5, 10, 15, 20%) were prepared by mixing LPMC with dibutyltin dilaurate (catalyst), water (blowing agent) and polyphenylmethane polyisocyanate (PMDI). The authors noted that the mechanical properties could be enhanced upon the inclusion of up to 15% LPMC. Both Young’s modulus and compression stress exhibited a two-fold increase (from 0.51 and 11.34 MPa to 1.46 and 31.56 Mpa, respectively). At higher levels, though, deterioration of mechanical properties was revealed.

The TGA revealed pure PU foam to have a *T_initial_* value of 291 °C and a *T_max_* of 358 °C and left only ca. 14% char at 600 °C. Pure LPMC, on the other hand, displayed more complex degradation with two *T_max_* values (314 and 383 °C, corresponding to the cleavage of the phosphate ester bond and charring, respectively) and left behind 53.3% char residue up to 800 °C. Incorporation of LPMC led to a decrease in *T_initial_* with increasing content (from 287.6 to 282.0 °C for 5 and 20% LPMC, respectively), first stage *T_max_* values similar to that of pure PU foam and second stage *T_max_* values of 406.1, 407.4, 408.2, and 408.5 °C (for 5, 10, 15, and 20% LPMC content). As expected, the char residue formation increased upon increasing LPMC content: 17.2, 22.2, 24.4, and 27.7 wt % for each sample. FR properties were measured as well. LOI values for pure PU and PU foams containing 5, 10, 15, and 20 wt % LPMC were 18.9, 23.4, 24.8, 26.7, 28.3%. Advantageously, samples containing 15 and 20 wt % LPMC reached V-1 rating without dripping in UL-94 tests. 

The use of covalently-modified lignin as an FR additive to other synthetic polymers has been reported as well. Recently, Bourbigot’s [64] laboratory showed that kraft lignin could easily be phosphorylated through reaction with phosphorus pentoxide (P_2_O_5_). The resulting material (P-Lig) was then used to prepare FR ABS composites. Thermogravimetric analysis (TGA) was utilized to study the thermal behavior of these materials. Under air it was revealed that pure ABS exhibited *T*_5%_ and *T_max_* values of 325 and 405 °C. Upon the inclusion of 30 wt % P-Lig, these values could be decreased to 306 and 355 °C. Char residue was shown to be relatively low: 1.6% for pure ABS and only a two-fold increase with 30 wt % P-Lig. This could be increased upon analysis under inert atmosphere (ca. 17.1%). Calorimetry was further employed to study the flammability of each sample. Pure ABS exhibited PHRR and THR values of 482 kW/m^2^ and 72 MJ/m^2^. Upon the incorporation of 30 wt % P-Lig these values were decreased to 202 kW/m^2^ and 58 MJ/m^2^, indicating higher flame retardance. Furthermore, evolution of potentially toxic gases could be dramatically reduced. For instance, inclusion of 30 wt % P-Lig resulted in a 47% decrease in CO_2_ generation, 30% decrease in CO generation, 47% decrease in NO formation, and 27% decrease in HCN evolution. 

**Figure 18:**
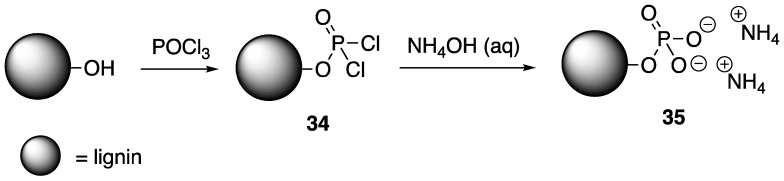
Preparation of **35**.

As noted earlier, Laoutid and coworkers [38] showed that biodegradable PLA could be instilled with high FR properties through melt-blending with a combination of phytic acid and lignin. In 2016, the same group illustrated the use of P and N-derivatized lignin as an FR for PLA [65]. The modification of kraft and organosolv lignin was performed through esterification with phosphorus chloride to form intermediate **34**, followed by reaction with ammonium hydroxide to provide modified lignin **35** (Figure 18). **35** was used to prepare FR PLA composites using melt processing.

The authors studied the thermal properties of the resulting composites using TGA. Kraft and organosolv lignin samples decomposed to leave 50 and 48% residue at 800 °C. The incorporation of P and N groups (**35**) increased these values to 58 and 60% for kraft and organosolv, respectively. This indicates that the modified materials could potentially serve as FR additives. PLA composites of 20 wt % lignin and 20 wt % **35** were then analyzed. Pristine PLA exhibited a 10% weight loss (*T*_10%_) at 330 °C while composties of kraft and organosolv lignins were 225 and 275 °C, respectively. The inclusion of lignin promotes char formation in which PLA–lignin composites left behind ca. 15% residue at 600 °C. The PLA composites of chemically-treated lignin (**35**), however, exhibited thermal properties similar to those of pure PLA, but promotes more char formation (ca. 13%) than PLA. Cone calorimetry was utilized to study the FR properties of these composites. It was found that incorporation of un-modified lignins reduced the PHHR values of PLA by 21 (kraft) and 33% (organosolv), due to protective char formation. Interestingly, in the case of modified lignins **35**, PHHR values could be dramatically reduced only in with kraft lignin. For both kraft and organosolv **35** samples, TTI could be increased from two-fold (compared to unmodified lignin composites) to values similar to pristine PLA. This resistance to ignition is due to the presence of the ammonium groups. Furthermore, composites prepared from **35** samples reached V0 ratings in UL-94 burn tests.

Other groups have used the excellent char-forming capability of lignin to prepare FR PLA as well. That same year, Cayla et al. [66] prepared an intumescent system from kraft lignin (LK) and ammonium polyphosphate **8**. They utilized twin-screw extrusion to prepare FR PLA composites of varying LK and **8** content. Selected data for thermal behavior measured using TGA is summarized in Table 11. Samples containing 10 wt % **8** were impervious to ignition in UL-94 flammability tests and exhibited no melt dripping. Samples composed of 5 and 10 wt % LK and 5 and 10 wt % **8** exhibited V0 rating. 

**Figure 19:**
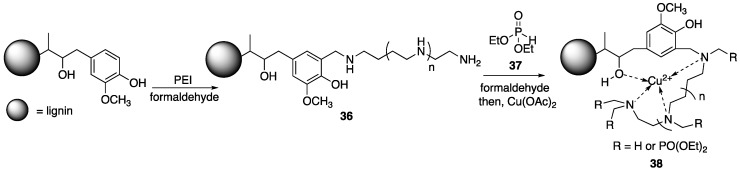
Synthesis of metalated lignin, **38**.

Wood–plastic composites (WPC) have found wide applicability in many industrial settings. Because of its cost and abundance, polypropylene (PP) is often utilized in this regard [67,68]. However, these composites are highly flammable. Recently, Song and Yu et al. [69] were able to fabricate WPC containing FR additives based off of covalently-modified lignin. To begin, the authors subjected purified alkali lignin to reaction with **7** in the presence of formaldehyde to provide **36** which was then subjected to reaction with diethylphosphite **37** and formaldehyde (Figure 19). This material was then metalated with copper(II)acetate (Cu(OAc)_2_) to form **38**. The reason for the last step is that it has been shown that the presence of metal ions can have some synergistic effects on FR properties (i.e., enhancing char formation). Wood–plastic composites, of 76.5 wt % PP and varying **38** content, were prepared via melt compounding.

The flammability properties of each composite were tested using UL-94 burn tests. Wood–plastic composites containing no lignin had no rating while composites containing 15 wt % pristine lignin were rated at V-2. Wood–plastic composites consisting of 10 wt % **38** was rated at V-1, indicating better flame retardance. Cone calorimetry was employed to further study the flame retardance of these materials. Wood–plastic composites with no added lignin showed PHRR and THR values of 595 kW/m^2^ and 93.9 MJ/ m^2^. Inclusion of 15 wt % lignin decreased these values to 580 kW/m^2^ and 85.3 MJ/m^2^ while using 15 wt % **38** further diminished these values to 470 kW/m^2^ and 70.2 MJ/m^2^. Further, WPC consisting of either lignin or **38** resulted in decreased smoke production. For example, the TSR (total smoke production rate) for WPC with no lignin was 12.5 m^2^/m^2^, while the addition of 15 wt % lignin or **38** decreased the TSR to 10.9 and 9.17 m^2^/m^2^, respectively.

These same groups later used similar procedures to prepare FR composites based on polybutylene succinate (PBS) [70]. In this report, **36** (Figure 19) was metalated with zinc(II)acetate (Zn(OAc)_2_) to form PNZn-lig **39**. Analysis by TGA in air revealed that **39** produced higher char residues (30 wt % at 800 °C) than did pristine lignin (25 wt %), indicating some enhancement of the FR properties. Pure PBS matrix exhibited *T_init_* and *T_max_* values of 354 and 410 °C. Incorporation of only 2.5 wt % lignin increased the *T_init_* (365 °C), but not the *T_max_*. The increase in *T_init_* is due to the early formation of protective char. Although the incorporation of 10 wt % **39** resulted in earlier *T_init_* values, it did leave the most char upon decomposition at 500 °C (10%). Similar to their findings with WPC, cone calorimetry revealed that the inclusion of modified lignin resulted in higher levels of flame retardance. For instance, PBS exhibited PHRR and THR values of 500 kW/m^2^ and 18.8 kJ/g. The incorporation of only 2.5 wt % lignin decreased these values to 416 kW/m2 and 21.6 kJ/g while 2.5 wt % **39** incorporation exhibited values of 447 kW/m^2^ and 14.4 kJ/g. These values could be further decreased upon increasing **39** content. For example, at 10 wt % **39**, PHRR and THR were 244 kW/m^2^ and 6.1 kJ/g. All composites of lignin and modified lignin ceased droplet formation during combustion and resulted in higher levels of char formation (10 wt % **39** left ca. 55% residue). Additionally, these materials served to inhibit smoke production. The PBS matrix showed a TSR value of 274 m^2^/m^2^ while the composite consisting of 10 wt % **39** was 125 m^2^/m^2^.

**Figure 20:**
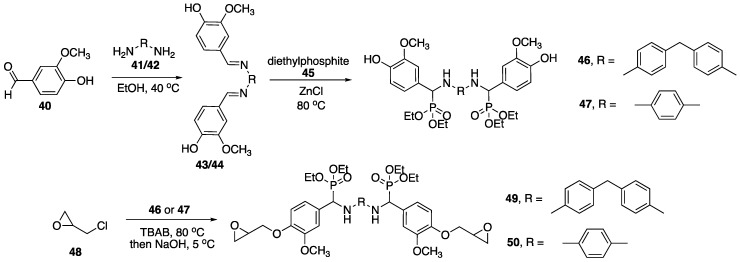
Preparation of **46**, **47**, **49**, and **50**.

Because of its low cost and abundance in nature, the use of lignin as a carbon feedstock has been recognized. In fact, it can be used to prepare numerous small molecules. Vanillin **40** (the common component of vanilla) is one of the processes that has been commercially utilized and is presumably much cheaper and efficient than extraction from other sources [71]. Epoxy resins are among some of the most industrially-important polymeric materials. They are used in a wide variety of applications, but can be limited by their high levels of flammability. It is for this reason that the development of FR epoxy resins is crucial. In 2017, Ma, Zhu, and coworkers [72] reported on the preparation of FR epoxy resins based off of chemically-modified vanillin (produced from lignin). The synthesis started with the functionalization of **40** with **41** or **42** to form either **43** or **44**. This was followed by the addition of diethylphosphite **45** in the presence of ZnCl to afford either **46** or **47** (Figure 20). These products were then subjected to reaction with epichlorohydrin **48** followed by base to provide epoxides **49** and **50**. Subsequent curing with **46** (DDM) at 200 °C lead to vanillin-based epoxy resins. 

Upon isolations, these resins were then subjected to UL-94 burn tests. The LOI values resin prepared from **46** and **49** (**49**–**46**) and **50**–**46** were 31.4 and 32.8%, respectively. These were both rated at V0 while control resin (DGEBA-**46**) exhibited no rating and had an LOI value of 24.6%. Further, **49**–**46** and **50**–**46** left behind considerable char residue upon combustion (35.8 and 41.2%). Analysis by TGA revealed that both **49**–**46** and **50**–**46** exhibited lower *T_init_* values compared to DGEBA-**46**, as is consistent with P-containing FR. Because of the increased char formation, **49**–**46** and **50**–**46** exhibited higher *T_max_* values than control resin. As expected, control DGEBA-DDM underwent complete decomposition under air while **50**–**46** left almost 60% char residue at 700 °C.

### 2.7. β-Cyclodextrin

**Figure 21:**
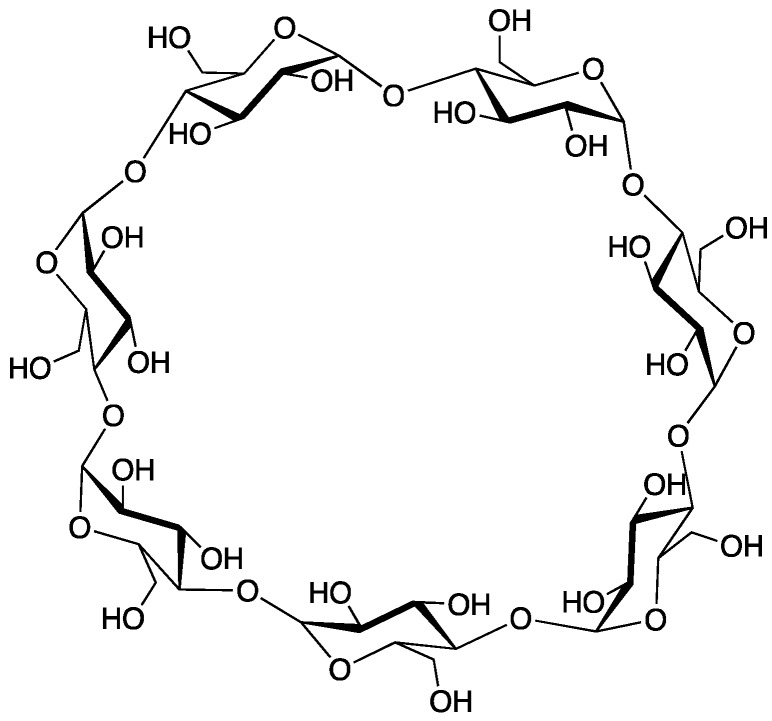
Molecular structure of *β*-cyclodextrin (*β*-CD).

Cyclodextrins are macrocyclic structures comprised of multiple glucose units; wherein those constructed of **6**, **7**, or **8** (known as *α*-, *β*-, or *γ*-cyclodextrin) are the most common. These compounds are synthesized via enzymatic degradation of starch and their existence has been known for quite some time [73]. Because of its smooth decomposition and high char yield, *β*-cyclodextrin (*β*-CD, structure shown in Figure 21) has attracted much interest as an FR additive [74,75,76].

**Figure 22:**
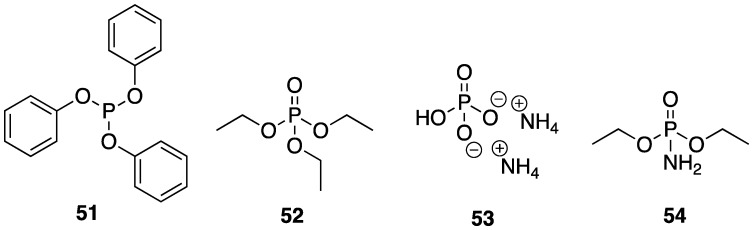
Molecular structures of P-species used.

Alongi and coworkers [77] described the use of *β*-CD as a dual action FR additive in which it serves as an IFR material as well as a nanofiller. The authors described the preparation of *β*-CD “nanosponges” (NS) through cross-linking *β*-CD units through carbonate linkages using simple mechanical grinding. These porous systems serve the purpose of trapping low molecular weight phosphorus-containing compounds (P), acting as a synergistic FR additive. The P species used were triphenyl phosphite **51**, triethyl phosphate **52**, ammonium polyphosphate **8**, dibasic ammonium phosphate **53**, and diethyl phosphoramidate **54** (Figure 22). These NS–P complexes were prepared through further grinding with various P species. The EVA composites with these materials were then prepared via melt-blending. The authors studied the flammability properties of the resulting composites using standard UL-94 tests. Pristine EVA burned completely and rapidly and was not classifiable. Ratings of V2 could be achieved upon the incorporation of 5, 10, and 15 wt % NS-**52** and 15 wt % NS complexes of **8**, **53**, and **54**. The behavior of these materials was further probed using cone calorimetry. Pristine EVA exhibited HRR value of 582 kW/m^2^. This value could be decreased upon the addition of NS–P complexes. For instance, the HRR of 10 wt % NS-**52** was 177 kW/m^2^ while 15 wt % NS-**53** was 177 kW/m^2^ and **54** was further decreased to 151 kW/m^2^. The PHRRs were dramatically affected and could be decreased from 1509 kW/m^2^ (for EVA) to 254 kW/m^2^ for 15 wt % NS-**54**.

A couple of years later, the same group utilized these NS materials as synergistic FR additives for PP, LLDPE, and PA6 [78]. The NS–phosphorus complexes were prepared using mechanical grinding of NS and either **52** or **8**. The resulting complexes were incorporated into the polymer matrix through mixing with PP, LLDPE, or PA 6 at elevated temperatures. The TGA revealed the effect that the NS–P complexes played on the thermal stability of the materials. Pristine PP exhibited 10% weight loss temperature (*T*_*onset*10%_) of 430 °C. Incorporation of the NS (10 wt %) alone lowered this to 406 °C. This effect is due to the hydroxyl groups catalyzing the decomposition of the polymer. Values of 424, 439 °C were obtained for PP composites of NS-**52** (7 wt % NS, 3 wt % **52**) and **8** (7.5 wt % NS, 7.5 wt % **8**) complexes, respectively. A similar effect was observed for LLDPE. It was argued that, in general, the NS–P complexes offered enhanced stability of polyolefins through the dehydration of *β*-CD moiety through the generation of phosphoric acid. The generation of water favored char formation. PA6 exhibited a *T*_*onset*10%_ of 411 °C. Composites with NS-**52** (7 wt % NS, 3 wt % **52**) showed values of 334 °C and 368 °C for NS-**52** complex of 10 wt % NS and 5 wt % **52**. The stability of the PA 6 is different than the polyofefins due to decomposition of the polymer during phosphoric acid generation. Even though TGA hinted at enhanced stability for these materials, char residues at temperatures of up to 700 °C were relatively low; the highest value was 8.6 wt % for the PA 6 composite of NS-**52** (10 wt % NS and 5 wt % **52**).

Cone calorimetry was again employed to explore the flame-retardant properties of these composites. Pristine PP exhibited PHRR and THR values of 1541 kW/m^2^ and 90 MJ/m2. The values of composites of 7 wt % NS and 3 wt % TEP were 1529 kW/m2 and 93 MJ/ m^2^. These values could be improved to 839 kW/m^2^ and 90 MJ/m^2^ upon the incorporation of 10 wt % NS and 5 wt % **52**. Composites of LLDPE exhibit different properties. The incorporation of both NS and NS–P complexes, in general, increased PHRR and THR. Incorporation of 15 wt % NS-**52** or **8** lowered these values, but produced substantial amounts of smoke. Unfortunately, incorporation of these composites did not result in any observable FR properties to PA6.

Considering that metal oxides and salts influence polymer degradation and char formation [79], Zhu’s [80] laboratory explored their incorporation into *β*-CD for FR polymer additives. The studied the effect such complexes have on the thermal stability and FR properties of poly(vinyl alcohol) (PVA), an important industrial polymer. The synthesis of the metalated CD (MC) complexes involved reaction of *β*-CD with maleic anhydride followed by formation of cross-linked metal complexes through the inclusion of hydroxide salts of Mg, Ca, and Ba to form MC–Mg, MC–Ca, and Mg–Ba, respectively. These synergists were incorporated into a PVA-**8** mixture and samples were prepared using by solution casting. The authors first reported the UL-94 results for such systems. Ratings of V0 could be reached with 15 wt % FR and 0.1–0.5 wt % metal MC (Mg, Ca, or Ba) with 15 wt % **8** was suitable to obtain this rating. Analysis of the thermal stabilities by TGA revealed that incorporation of **8** and *β*-CD reduced the initial degradation temperatures (*T*_5%_) compared to PVA; this is due to the decomposition of **8**. However, inclusion of metal salts increased this by approximately 16 °C; this is due to the stabilization of the metal ions. The composites containing metal ions also left behind higher levels of char residue. It was revealed that, although the initial degradation stage of these composites was lower than pristine PVA, the second and third stages were delayed.

**Figure 23:**
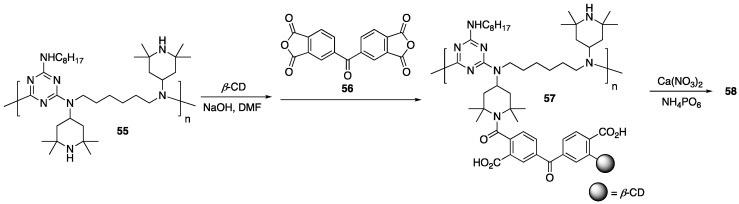
Preparation of **58**.

More recently, Vahabi, Shabanian, and coworkers [81] developed a new FR system based on *β*-CD, hydroxyapatite, and *N*-rich polymer, SABO **55**, which is commercially available. The FR additive was prepared by first mixing *β*-CD, **55**, and NaOH in DMF followed by addition of **56**. This material (**57**) was subsequently reacted with hydroxyapatite (formed in situ from NH_4_PO_6_ and Ca(NO_3_)_2_ to form the FR additive **58** (Figure 23). Composites of **58**-PLA (as well as **58**-PLA-**8**) were prepared by melt mixing. Once prepared, thermal stabilities were studied using TGA. These analyses revealed that the incorporation of **58** slightly affected the *T*_5%_ values, where pure PLA exhibited a value of 335 °C while the PLA/**58** was 334 °C. Inclusion of **8** further decreased this value to 323 °C. Whereas pure PLA decomposed to leave no char residue at 800 °C, pure **8** and **58** yielded 21 and 59%, respectively. PLA/**58**, PLA/**8**, and PLA/**58**/**8** composites yielded values of 12, 15, and 11%, respectively. Although **58** alone yielded far higher char residue than **8**, the fact that the PLA/**58** composites left less char than the PLA/**8** composite is not discussed. In any case, this data is indicative of possible enhancement of FR properties. To further probe this, cone calorimetry was employed to further study the fire behavior, the data is shown in Table 12. 

As can be seen from the data, inclusion of **58** showed to have little effect on the fire behavior of PLA, while **8** exhibited modest reduction in PHRR (from 365 to 301 kW/m2). A synergistic effect was observed, however, with PLA composites of both **8** and **58**, this value could be cut almost in half. Further, the THR values were also reduced two-fold and an increased char residue (40 wt %) was observed.

**Figure 24:**
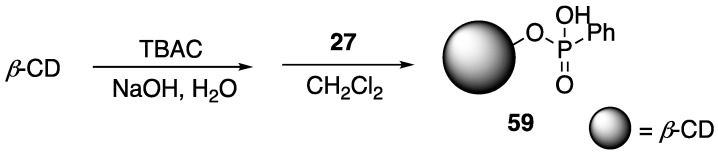
Reported preparation of **59**.

That same year, Fang and coworkers [82] described the covalent modification of *β*-CD with phenyl phosphonic acid dichloride **27** to form new phospholidpidated *β*-CD **59** (Figure 24). The reasoning behind this was the use of a P-containing *β*-CD would favor direct generation of phosphoric acid upon combustion. Modified *β*-CD **59** was then used as an FR additive to PLA and as a synergist in PLA-**8** systems. Preparation of PLA composites were conducted through melt blending. The thermal stabilities of the samples were then analyzed using TGA. First, the authors compared the thermal behaviors of **59** and *β*-CD. *β*-cyclodextrin alone exhibited *T*_5%_ of 316 °C and left ca. 11 wt % char residue at 700 °C. Modified *β*-CD **59** exhibited a ca. 60 °C decrease in *T*_5%_ (due to decomposition of the P-moiety), but left behind app. 30 wt % char residue. This suggested that **59** may act as a competent FR additive. Composites of PLA and **59** were then studied. Pristine PLA exhibited a *T*_5%_ value of 351 °C. Incorporation of 30 wt % **59** lowered the *T*_5%_ by 66 °C. Additionally, this composite decomposed to leave higher char residue, albeit only 6.6 wt %. Synergistic effects of **59** with **8** were subsequently probed but first the authors found that composites of PLA with **8** (30 wt %) lowered the *T*_5%_% to 339 °C. Even though **8** promoted char formation, this char was unstable and further decomposed. The PLA composites with 30 wt % **8**–**59** (of varying ratios) led to increased char residue yields. Values close to 20 wt % could be achieved through the incorporation of 30 wt % **8**–**59** (2:1) and 30 wt % **8**–**59** (1:1). The authors deduced that this was from further esterification of hydroxyl groups of PCD with phosphoric acid generated from **8** decomposition. 

The fire behaviors of these systems were then studied. It was shown that the incorporation of **8** and **59** could have dramatic effects on LOI. These values could be increased from 19.7% (pure PLA) to 19.9% (PLA–**59**) and 33% (PLA–**8**), all the way up to 42.6% (PLA–**8**/**59** (5:1). Furthermore, composites composed of 11:1, 5:1, 2:1, and 1:1 mixture of **8** and **59** were rated V0 in UL-94 tests.

## 3. Conclusions

The advent and commercialization of synthetic polymers has played a tremendous role in advancing our modern lives. The explosive growth of the development of new polymeric materials, that started in the twentieth century, shows no signs of slowing. Continued advances in polymer science translate to advances in vital fields such as engineering and medicine, as well as luxuries like cosmetics and apparel. However, there are downsides to further surrounding ourselves with these synthetic materials, one of which is their relatively high levels of flammability. However, if the trend in Figure 1 is any indication, discovering and inventing ways to prevent this flammability will also continue to grow. Furthermore, despite repeated environmental blunders by the current administration in the United States, the scientific community (as well as our society in general) is becoming more and more cognizant of the negative environmental effects of chemical research and production. For this reason, the implementation of the green chemistry principles will continue to guide our efforts in developing more sustainable FR. Paramount to this endeavor will be the increased use of bio-based materials, which can be obtained with less of a reliance on finite petroleum reserves than typical synthetic processes. This renders their use inherently more sustainable. Selected examples of bio-based FR and their respective PHRR values discussed in this review have been shown in Table 13. The examples described in this review highlight many of the positive discoveries that have been made in this field. There is still a long way to go, though. One detriment may be incompatibility between a low-molecular weight, hydrophilic FR, and a hydrophobic polymer that could potentially cause leaching of the FR. This problem could be solved through covalent attachment of the FR in question, but could also lead to changes in the mechanical properties of the material. This is obviously an issue that will come up as the field progresses. Although many of the bio-based FR discussed can be obtained in large quantities, this is not always the case. In fact, the extraction of such materials can solve as a bottle neck. More efficient, high throughput technologies for obtaining these materials will also need to be developed. 

## Figures and Tables

**Table 1:** Mechanisms of flame retardancy based on phase and example of each.

Phase	Method	Example
Condensed	formation of protective char	P-containing FR
Vapor	termination of free radicals involved in combustion	halogenated FR

**Table 2:** FR data for epoxy-clay composites.

Entry	TTI ^1^ (s)	PHRR ^2^ (kW/m^2^)	THR ^3^	TTPHRR ^4^	FIGRA ^5^	Residue (%)	LOI ^6^ (%)
E20C5	6	407	47	160	2.5	18.5	19
E20C5 coated	3	335	61	175	1.9	22.5	21
E20C5T2	7	370	51	165	2.2	21.1	20
E20C5T2 coated	3	319	62	185	1.7	21.6	21

^1^ time to ignition; ^2^ peak heat release rate; ^3^ total heat release; ^4^ time to peak of heat release rate; ^5^ fire growth rate; ^6^ limiting oxygen index.

**Table 3:** Vertical flame test data.

Sample	Burning Rate (mm/s)	Afterflame (s)	Char Length (mm)
Nylon 66	12.5	18	127
Nylon 66 (coated with tannic acid)	10.2	25	127
Nylon 66 (coated with **4**)	6.4	0	76

**Table 4:** Thermal analysis of polypropylene-polyelectrolyte (PP–PEC) composites.

Sample	*T*_5%_ (°C)	*T_max_* (°C)	Residue (%)
**7**	247	345	4
**5**	253	308	78
PEC	241	321	61
PP	271	354	1
PP-5% PEC	263	357	3
PP-10% PEC	256	368	6
PP-20% PEC	263	379	11
PP-20% **7**	287	392	1
PP-20% **5**	260	321	10

**Table 5:** Cone calorimetry data and UL-94 results for PLA–**5**–lignin composites.

Entry	TTI (s)	PHRR (kW/m^2^)	THR (MJ/m^2^)	Residue (%)	UL-94
PLA	87	390	90	0	NC
20% **5**	71	270	74	13	V-2
20% Kraft	37	310	71	17	NC ^1^
20% Organosolv	26	260	67	19	NC
15% **5**/5% Kraft	77	285	74	13	V-2
10% **5**/10% Kraft	43	220	74	12	V-2
15% **5**/5% Organosolv	61	250	78	13	V-2
10% **5**/10% Organosolv	46	250	65	13	V-2

^1^ No classification.

**Table 6:** Vertical burn test data for FR polyester sol-gel composites prepared using layer-by-layer (LbL) assembly.

Sample	After Flame (s)	Char Length (mm)	LOI (%)	Burning Rate (mm/s)	Melt-Dripping (%)
Control	44.0	26.9	21.6	1.19	44.0
5 min soak	30.2	26.4	30.3	0.35	30.2
10 min soak	25.0	16.1	30.5	0.34	25.0
15 min soak	15.9	13.5	30.8	0.33	15.9
20 min soak	16.4	12.3	31.4	0.32	16.4

**Table 7:** Cone calorimetry data for poly(vinylchloride) (PVC)–**5**-metal composites.

Sample	TTI (s)	Char Residue (%)	LOI (%)	PHRR (kW/m^2^)	TSP (m^2^)
Control	13	1.68	24.9	329.67	42.20
Sn	14	14.68	30.3	213.75	19.51
Zn	14	17.07	29.7	225.25	23.16
Cu	10	18.93	29.3	181.77	15.77
Al	15	17.50	27.3	245.34	25.45

**Table 8:** Cone calorimetry data for PLA–**28** blends.

Sample	TTI (s)	PHRR (kW/m^2^)	Residual Mass (wt %)	THR (MJ/m^2^)
Control	68	418	1.6	70
PLA 2	68	394	2.5	69
PLA 4	78	396	3.1	67
PLA 6	74	388	3.5	66

**Table 9:** Flammability of PLA composites of **28**.

Sample (28:M-GO)	LOI (%)	UL-94
PLA	20	NR
PLA-3:0	33.6	V0
PLA-2.7:0.3	35.3	V0
PLA-2.4:0.6	36.0	V0
PLA-2.1:0.9	32.7	V2

**Table 10:** Flammability of polymers surfaced-treated with DNA.

Heat Flux (kW/m^2^)	Property	PP	PP–DNA	PET	PET–DNA	ABS	ABS–DNA	PA 6	PA 6-DNA
35	PHRR (kW/m^2^)	1300	629	973	563	1180	512	1036	562
	TTI (s)	15	156	82	274	26	344	19	330
50	PHRR (kW/m^2^)	1800	900	892	525	1479	433	1488	810
	TTI (s)	32	58	27	246	12	80	48	166

**Table 11:** Thermal properties of PLA composites.

Sample	*T_onset_* (°C)	*T_max_* (°C)	Char Residue at 500 °C (%)
PLA	330.7	370.5	1.1
LK	232.1	357.8	43.5
**8**	334.6	580.7	82.4
PLA-LK_5%_-**8**_%_	327.7	372.3	10.5
PLA-LK_10%_-**8**_%_	320.0	362.0	14.3

**Table 12:** Thermal properties of PLA composites of **58** and **8**.

Sample	PHRR (kW/m^2^)	THR (MJ/m^2^)	Char Residue (wt %)
PLA	365	89	0
PLA/**58**	365	73	5
PLA/**8**	302	65	15
PLA/**58**/**8**	185	45	40

**Table 13:** Selected examples of bio-based FR, polymeric material treated/prepared, and PHRR values.

Bio-Based FR	Polymeric Material	PHRR	PHHR of Virgin Material
tannic acid	epoxy	335 kW/m^2^	407 kW/m^2^
phytic acid	EVA/chitosan	552 W/g	801 W/g
isosorbide	PLA	744 kW/m^2^	786 kW/m^2^
diphenolic acid	PLA	388 kW/m^2^	418 kW/m^2^
DNA	EVA	963 kW/m^2^	1588 kW/m^2^
lignin	PPE	405 kW/m^2^	1350 kW/m^2^
*β*-CD	EVA	254 kW/m^2^	1509 kW/m^2^
